# Modular synthesis of 2-furyl carbinols from 3-benzyldimethylsilylfurfural platforms relying on oxygen-assisted C–Si bond functionalization

**DOI:** 10.3762/bjoc.18.131

**Published:** 2022-09-16

**Authors:** Sebastien Curpanen, Per Reichert, Gabriele Lupidi, Giovanni Poli, Julie Oble, Alejandro Perez-Luna

**Affiliations:** 1 Sorbonne Université, CNRS, Institut Parisien de Chimie Moléculaire, IPCM, 4 Place Jussieu, 75005 Paris, France

**Keywords:** biomass, copper, cyclic siloxanes, 2-furyl carbinols, silicon

## Abstract

3-Silylated furfurals, readily prepared in three steps from biomass-derived furfural and 5-methylfurfural, are converted into 3-silylated 2-furyl carbinols upon condensation with organomagnesium or organolithium reagents. The hydroxy unit of the carbinol adducts can be exploited to promote C3(sp^2^)–Si bond functionalization through intramolecular activation. Two approaches were contemplated for this purpose. Activation by alkoxides of the C3–SiEt_3_ or C3–SiMe_2_*t*-Bu bonds was ineffective. Conversely, treatment of the *C*3-benzyldimethylsilyl-appended derivatives with tetrabutylammonium fluoride led to cyclic siloxanes, which revealed to be competent donors for copper-catalyzed cross-coupling reactions, such as arylation reactions catalyzed by Pd_2_(dba)_3_/CuI, as well as allylation and methylation reactions catalyzed by CuI⋅PPh_3_. *C*3-Benzyldimethylsilyl-appended furfurals are thus introduced as versatile platforms, providing a modular access to 3-substituted 2-furyl carbinols from renewable feedstock.

## Introduction

**Scheme 1:**
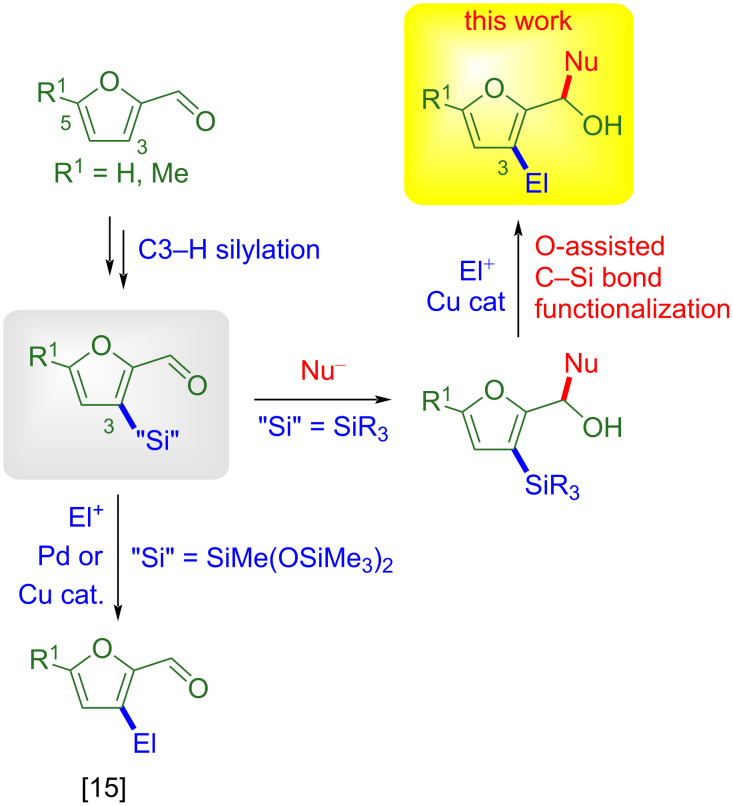
C3–Si bond functionalization of biomass-derived 3-silylated furfural platforms.

Progress towards the use of nonedible renewable feedstock to replace fossil resources as starting material for high-value chemicals is an important endeavor of modern synthesis [1–3]. As part of this effort, exploitation of furfural and corresponding derivatives attracts continuous attention [4–7]. In this area, we have actively investigated catalytic C–H activation reactions [8–14], and as part of this work, we have recently developed an iridium-based protocol for the catalytic C3–H silylation of furfurylimines [15]. This method allows to install a C–Si bond poised for further functionalization on the furfural unit, and thereby leads to synthetic platforms useful to access elaborated furans. This prospect was demonstrated with platforms relying on the SiMe(OSiMe_3_)_2_ unit, which were readily converted through Pd- or Cu-catalyzed electrophilic substitution reactions into an array of furfurals decorated at C3 with carbon- or heteroatom-containing substituents (Scheme 1). Conversely, all of our subsequent efforts to achieve cross-coupling reactions from C3-silylated furfurals with triorganosilane units have failed so far.

2-Furyl carbinols represent a useful class of furanic synthetic intermediates that have given rise to a number of synthetically relevant transformations, such as the Piancatelli and aza-Piancatelli reactions [16–17] or the Achmatowicz rearrangement [18]. Addition reactions of nucleophiles to the C–O double bond of furfurals represent an obvious synthetic approach to 2-furyl carbinols. We reasoned that for carbinols derived from *C*3-triorganosilyl-substituted furfurals, the OH unit could be exploited to assist C–Si bond activation [19]. Thereby, *C*3-triorganosilyl-substituted furfurals could be suitable platforms to develop a two-step modular approach to 3-substituted 2-furyl carbinols, entailing nucleophilic addition to the aldehyde function and oxygen-assisted electrophilic substitution of the C–Si bond (Scheme 1).

## Results and Discussion

### Synthesis of 3-silylated 2-furyl carbinols

**Scheme 2:**
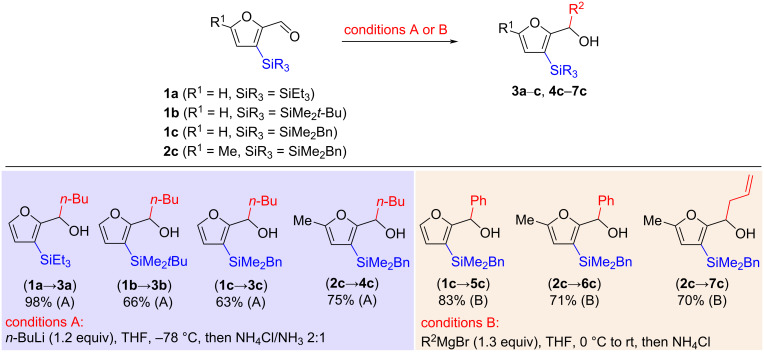
Preparation of 3-silylated 2-furyl carbinols.

C3-silylated furfurals **1a**–**c** and **2c** are accessible from furfural or 5-methylfurfural [20], respectively, according to our previously reported protocol for selective catalytic C3 silylation [15]. The addition of organolithium [21] or Grignard reagents [22–23] to these substrates was uneventful and allowed for the preparation of 2-furylalkyl (see **3a**–**c**, **4c**), -aryl (see **5c**, **6c**), and -allyl carbinols (see **7c**) having furan rings with various triorganosilyl substituents at C3 in a synthetically useful yield and on an appropriate scale (Scheme 2).

With the 3-silylated 2-furyl carbinol substrates at hand, we then considered C–Si bond activation strategies relying on the assistance of the oxygen atom to promote electrophilic substitution reactions with carbon electrophiles.

### C3–Si bond functionalization through intramolecular activation by alkoxides

**Scheme 3:**
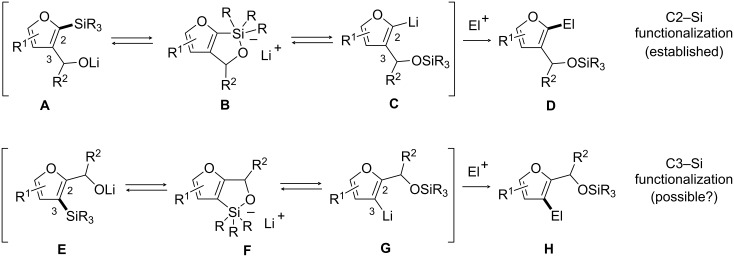
C–Si bond functionalization of 2,3-disubstituted furyl carbinols by 1,4-silyl migration.

We first contemplated the possibility to promote C3–Si bond functionalization through intramolecular activation by alkoxides [15]. It was reported that lithium alkoxides **A** undergo 1,4-silyl migration (Brook rearrangement) to generate C2-lithiated furans **C**, which in turn can react in the presence of electrophiles to deliver product **D**, resulting from the overall C2–Si functionalization (Scheme 3, top) [24–25]. The key to the viability of this process is the formation of pentavalent silicon intermediate **B**. This suggested that a related pentavalent intermediate **F** could be similarly accessible from **E**, and thus affording the C3-lithiated furan derivative **G** upon 1,4-silyl migration as well as the electrophilic substitution product **H** in the presence of an appropriate electrophile (Scheme 3, bottom).

**Scheme 4:**
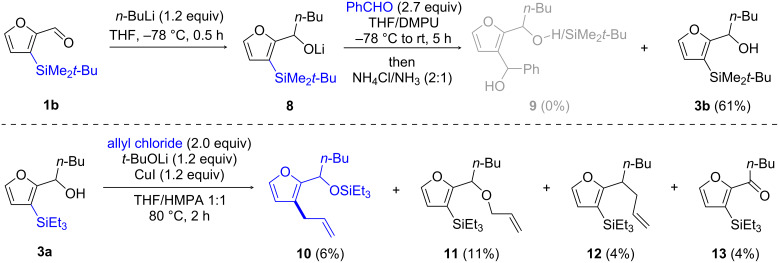
Attempts of C3–Si bond functionalization promoted by intramolecular activation via alkoxide.

However, treatment of aldehyde **1b** with *n*-BuLi, followed by addition of benzaldehyde in THF/DMPU [25], afforded only the addition product **3b** without any detectable formation of product **9**, expected from 1,4-silyl migration/electrophilic substitution of **8** (Scheme 4, top).

Conversely, treatment of alcohol **3a** with *t*-BuOCu in the presence of allyl chloride, according to conditions developed by Takeda et al. for the reaction of γ-trimethylsilyl-substituted allylic alcohols [26–27] or *ortho*-silylated aryl carbinols [28], did provide C3-functionalized compound **10**, albeit in very low yield (6%) and along with the O-allylated product **11** (11%), as well as products **12** (4%) and **13** (4%) [29] (Scheme 4, bottom). In spite of extensive experimentation to find better conditions, this result could not be improved.

**Scheme 5:**
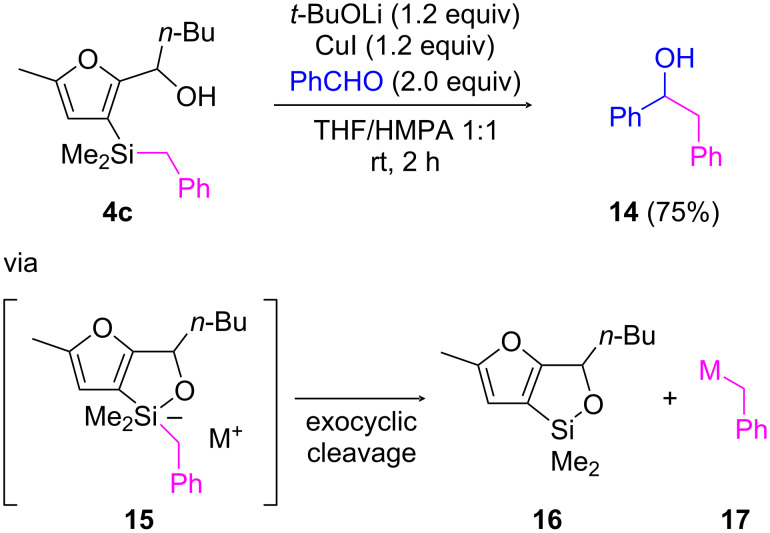
Alkoxide-promoted cyclic siloxane formation from 2-[(3-benzyldimethylsilyl)furyl] carbinol **4c**.

Nevertheless, important information came from an experiment involving furyl carbinol **4c**, having a benzyldimethylsilyl unit (Scheme 5). Here, treatment with *t*-BuOCu⋅LiI in THF/HMPA in the presence of benzaldehyde led to the formation of adduct **14** (in 75% yield), which arose from the addition of a benzyl carbanion **17** to benzaldehyde. The generation of such a nucleophile strongly suggests the formation of pentavalent silicon intermediate **15** [27], which then produced a (stabilized) benzylic carbanion (by exocyclic cleavage) rather than a 3-furanyl anion (by endocyclic cleavage). As a consequence, the failure to promote C3–Si activation of intermediates **E** (SiR_3_ = SiEt_3_ or SiMe_2_*t*-Bu) according to our initially envisaged scenario depicted in Scheme 3, did not seem to be related to the difficulty to form pentavalent silicon intermediates **F**. Instead, the reason was probably a too low intrinsic stability of the C3-lithiated furans **G**, which thwarted 1,4-silyl migration. Conversely, this experiment provided evidence that cyclic siloxanes (e.g., **16**), which are potential nucleophilic partners for cross-coupling chemistry [30–31], could be accessed from 2-[(3-benzyldimethylsilyl)furyl] carbinols.

### C3–Si bond functionalization through siloxane formation

**Scheme 6:**
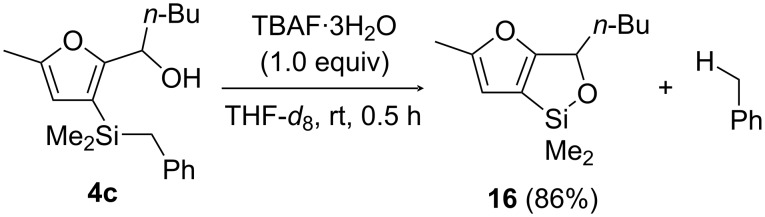
TBAF-promoted cyclic siloxane formation from 2-[(3-benzyldimethylsilyl)furyl] carbinol **4c**.

In an attempt to isolate cyclic siloxane **16**, we then considered a protocol reported by Anderson and co-workers to prepare cyclic siloxanes by debenzylation/cyclization of benzyldimethysilyl-substituted allylic alcohols [32]. To that end, carbinol **4c** was treated with TBAF⋅3H_2_O (1.0 equiv), which resulted in the formation of **16** in 86% yield (along with toluene, Scheme 6). Alike 5-membered cyclic dimethyl(alkenyl)siloxanes, **16** was found to be highly sensitive towards silica gel column chromatography, and all of our attempts to isolate it failed. For this reason, we went on to consider C3–Si functionalization strategies of alcohols **4c**–**7c** relying on the formation of cyclic siloxanes and subsequent in situ cross-coupling.

**Scheme 7:**
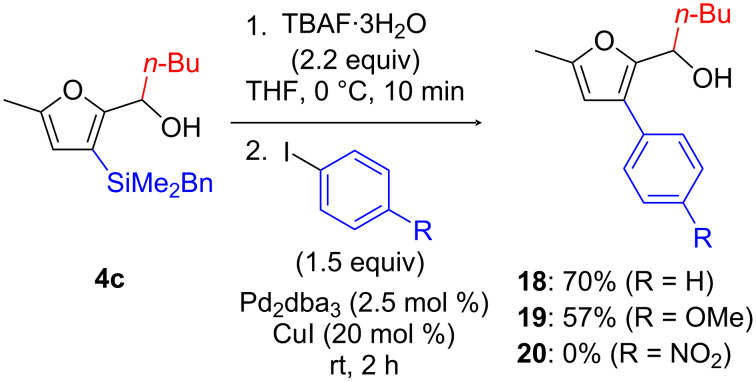
Pd-catalyzed arylation of 2-[(3-benzyldimethylsilyl)furyl] carbinol **4c**.

We first briefly investigated Pd-catalyzed arylation reactions (Scheme 7). Fluoride-promoted arylation reactions of benzyldimethyl(alkenyl)silanes have been reported, and it is established that they proceed through the cleavage of the benzyl moiety from the benzyldimethylsilyl groups, leading to either dimethylsilanols or cyclic siloxanes (as for substrates with pending hydroxy units) [32–35]. To the best of our knowledge, no analogous cross-coupling reactions from aryl- or heteroaryl-substituted benzyldimethylsilanes have been disclosed. We were thus delighted to find that using Pd_2_(dba)_3_ as precatalyst (2.5 mol %) in combination with CuI (20 mol %), cross-coupling between **4c** and iodobenzene was achieved, giving **18** in reasonably good yield (70%). 4-Iodoanisole could also be coupled (giving **19** in 57% yield), but not electron-deficient 1-iodo-4-nitrobenzene. At this point, it should be mentioned that treatment of aldehyde **2c** under the same reaction conditions led predominantly to decomposition products and only 5-methylfurfural (resulting from protodesilylation) was recovered in low yield (≈20%). Hence, intramolecular hydroxy activation seems decisive to obtain productive cross-coupling reactions with this system. Also, it is important to underscore that the copper cocatalyst was crucial for the success of this transformation as without, only protodesilylation products were obtained. This behavior is in contrast to that of dimethyl(alkenyl)siloxanes, which do not require copper to undergo cross-coupling [30,32].

This requirement for copper prompted us to test copper-catalyzed C(sp^2^)–C(sp^3^) cross-coupling reactions, as reported by Takeda et al., to achieve allylation reactions of benzyldimethyl(alkenyl)silanes [36]. Treatment of **4c** with methallyl chloride in the presence of TBAF⋅(*t*-BuOH)_4_ (2.4 equiv), CuI (1.5 equiv), and P(OEt)_3_ (1.5 equiv) in DMF at room temperature delivered, after hydrolysis, the cross-coupling compound **21** in 68% yield, along with 9% of protodesilylation product **22** (Table 1, entry 1). As indicated by Takeda et al., the amount and source of TBAF proved crucial: use of either 1.2 equiv of TBAF⋅(*t*-BuOH)_4_ instead of 2.4 equiv (Table 1, entry 2), or use of a (commercially available) 1-molar TBAF solution in THF (Table 1, entry 3), gave an increased amount of protodesilylation product **22** at the expense of **21**. By contrast, byproduct **22** was not observed reducing the reaction time from 24 h to 2 h, but the yield of product **21** remained the same (Table 1, entry 4). In spite of the satisfactory yield of **21**, these conditions were synthetically impractical as we were unable to conveniently separate the final product from the phosphite ligand. The use of a ligand for copper revealed to be necessary, given that the cross-coupling product **21** was obtained in only 40% yield (along with 23% of **22**) in the absence of P(OEt)_3_ (Table 1, entry 5). The solution was the replacement of triethylphosphite by triphenylphosphine, which allowed to suppress the formation of the protodesilylated product and to obtain product **21** in 80% yield (Table 1, entry 6). Similar results were obtained using the preformed complex CuI⋅PPh_3_ (1.5 equiv), so that compound **21** could be isolated in 65% yield (Table 1, entry 7). Importantly, the use of copper in a substoichiometric amount (20 mol %) was also suitable, allowing to isolate **21** in an even better 78% yield (Table 1, entry 9). It should also be mentioned that the use of THF instead of DMF as solvent led to worse results (Table 1, entries 7 and 8).

**Table 1:** Optimization of the reaction conditions for Cu-catalyzed methallylation of 2-[(3-benzyldimethylsilyl)furyl] carbinol **4c**.

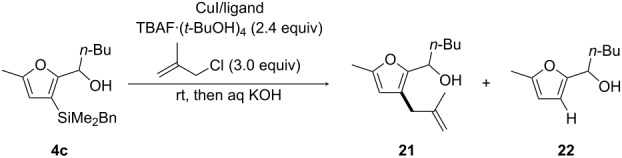					

entry	Cu source	ligand	conditions	**21**^a^ (%)	**22**^a^ (%)

1	CuI (1.5 equiv)	P(OEt)_3_ (1.5 equiv)	DMF, 24 h	68	9
2^b^	CuI (1.5 equiv)	P(OEt)_3_ (1.5 equiv)	DMF, 24 h	38	32
3^c^	CuI (1.5 equiv)	P(OEt)_3_ (1.5 equiv)	DMF, 24 h	41	20
4	CuI (1.5 equiv)	P(OEt)_3_ (1.5 equiv)	DMF, 2 h	68	0
5	CuI (1.5 equiv)	—	DMF, 2 h	40	23
6	CuI (1.5 equiv)	PPh_3_ (1.5 equiv)	DMF, 2 h	80	0
7	CuI⋅PPh_3_ (1.5 equiv)		DMF, 2 h	79 (65)^d^	0
8	CuI⋅PPh_3_ (1.5 equiv)		THF, 2 h	71	20
9	CuI⋅PPh_3_ (20 mol %)		DMF, 2 h	84 (78)^d^	0

^a^Yield measured prior to purification by ^1^H NMR analysis using Me_2_SO_2_ as internal standard. ^b^TBAF⋅(*t*-BuOH)_4_ (1.2 equiv) was used. ^c^TBAF (1 molar solution in THF, 2.4 equiv) was used. ^d^Isolated yield.

**Scheme 8:**
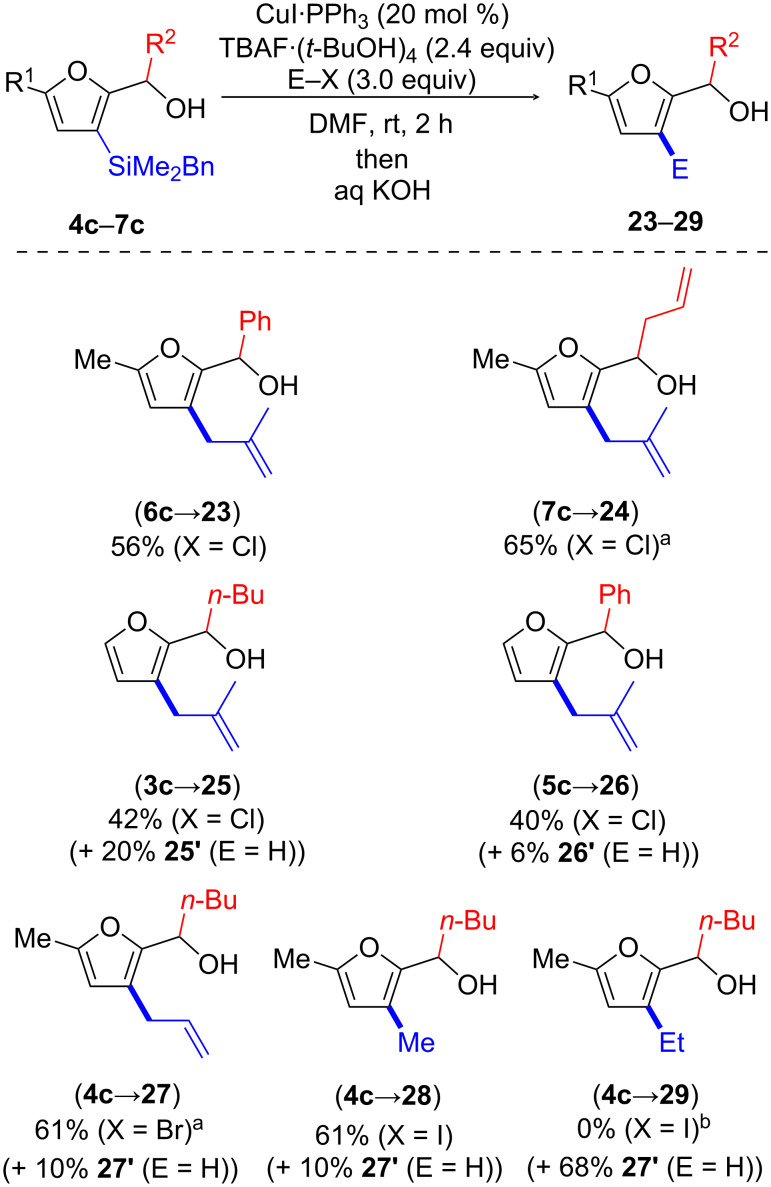
Cu-catalyzed allylation and methylation of 2-[(3-benzyldimethylsilyl)furyl] carbinols. ^a^CuI⋅PPh_3_ (120 mol %). ^b^CuI/P(OEt)_3_ (1.5 equiv), rt or 50 °C.

Investigating the scope of this transformation (Scheme 8), we established that the reaction performed well with other 5-methylfuryl carbinols. As such, products **23** and **24**, arising from phenyl- and allyl-substituted substrates **6c** and **7c**, respectively, were isolated in 56% and 65% yield, respectively. Somewhat lower yields were noted with butyl- and phenyl-substituted carbinols **3c** and **5c**, respectively, bearing C5-unsubstituted furan rings, which gave products **25** and **26** in 42% and 40% yield, respectively. Electrophiles other than methallyl chloride could also be used. C3-Allylation of substrate **4c**, leading to **27**, was achieved in 52% yield by reaction with allyl chloride and in a better 61% yield using allyl bromide. It should be mentioned that competing protodesilylation could not always be fully suppressed in the above described reactions. Furthermore, purification was troublesome for products **25**–**27**, which could only be isolated in the presence of the protodesilylated side products (see Supporting Information File 1 for details). We also contemplated the use of alkyl iodides as electrophiles. Methylation with methyl iodide was efficient, as shown through the preparation of **28** in 61% yield from **4c**. In contrast, higher alkyl iodides, such as ethyl iodide, failed to provide the alkylation product (i.e., **29**) and only protodesilylation was observed (even at 50 °C).

## Conclusion

In conclusion, we have shown that 3-silylated 2-furyl carbinols are readily accessible in three steps from furfural and 5-methylfurfural, and the hydroxy unit of these adducts can be used to promote C(sp^2^)–Si bond functionalization. Although intramolecular activation by alkoxides did not prove useful, C3–Si bond functionalization is achieved from benzyldimethylsilyl units upon siloxane formation in the presence of TBAF. Protocols for fluoride-promoted Pd/Cu-catalyzed arylation, as well as Cu-catalyzed allylation and methylation, have been developed. Overall, we have demonstrated that *C*3-benzyldimethylsilyl-appended furfurals are useful platforms that offer modular access to 3-substituted 2-furyl carbinols. This strategy represents a new, simple, and selective way to decorate the biomass-derived furan nucleus, allowing to reach synthetically relevant building blocks.

## Experimental

### Procedure for the addition of *n*-BuLi to C3-silylated furfurals (preparation of compounds **3a–c** and **4c**)

In a flame-dried round-bottom flask under argon was placed the appropriate C3-silylated furfural [15] and dissolved in freshly distilled THF (0.3 M). The solution was cooled to −78 °C, and then *n*-BuLi (1.2 equiv in hexane) was added dropwise. The reaction mixture was allowed to stir at −78 °C for 30 min and then quenched with aq saturated NH_4_Cl/NH_3_ 2:1 solution. Et_2_O was added, and the aqueous layer was extracted three times. The combined organic layer was washed with brine, dried over MgSO_4_, filtered, concentrated under reduced pressure, and purified by silica gel column chromatography.

### Procedure for the addition of Grignard reagents to C3-silylated furfurals (preparation of compounds **5c**, **6c**, and **7c**)

In a flame-dried round-bottom flask under argon was placed the appropriate C3-silylated furfural [15] and dissolved in freshly distilled THF (0.2 M solution). The solution was cooled to 0 °C, and then the Grignard reagent (1.3 equiv in Et_2_O) was added dropwise (the rate of addition was equal to, or lower than 0.125 mL/min). The mixture was allowed to stir at 0 °C for 1 h and then allowed to reach room temperature. Upon consumption of the starting material, the reaction mixture was quenched with saturated NH_4_Cl. CH_2_Cl_2_ was added, and the aqueous layer was extracted three times using CH_2_Cl_2_. The combined organic layer was dried over Na_2_SO_4_, filtered, concentrated under reduced pressure. and purified by silica gel column chromatography.

### Procedure for the Pd/Cu-catalyzed arylation of *C*3-benzyldimethylsilyl-substituted 2-furyl carbinol **4c** (preparation of compounds **18** and **19**)

A flame-dried Schlenk tube was charged with CuI (20 mol %) and Pd_2_(dba)_3_ (2.5 mol %) and heated gently under vacuum using a heat gun. In another Schlenk tube, the appropriate iodoarene (1.5 equiv) and **4c** (1 equiv) were dissolved in freshly distilled THF (0.63 mL). The solution was degassed in three freeze–pump–thaw cycles, and degassed anhydrous TBAF (1 M in THF, 2.2 equiv) was added. The mixture was allowed to stir for 10 min at 0 °C and transferred via cannula to the Schlenk tube containing the catalytic mixture. The resulting mixture was stirred for 2 h at rt, then filtered through a short pad of silica gel, and concentrated. The residue was purified by silica gel column chromatography.

### Procedure for the Cu-catalyzed allylation and methylation of *C*3-benzyldimethylsilyl-substituted 2-furyl carbinols (preparation of compounds **21** and **23–28**)

CuI⋅PPh_3_ [37] (20 or 120 mol %) was introduced to a flame-dried microwave vial, which was then placed under an argon atmosphere and sealed. In a Schlenk tube, the appropriate *C*3-benzyldimethylsilyl-substituted 2-furyl carbinol (0.3 mmol, 1 equiv) was dissolved in CH_2_Cl_2_ (1 mL), concentrated under reduced pressure, and placed under argon. DMF (3.0 mL) was then added, and the solution was degassed in three freeze–pump–thaw cycles. TBAF⋅(*t*-BuOH)_4_ [38] (402 mg, 0.72 mmol) was then added along with the corresponding electrophile (3 equiv). The mixture was stirred for 2–3 min at rt and then cannulated into the vial containing the copper complex. The mixture was stirred for 2 h at 30 °C and then quenched with aq KOH (6 M, 1 mL). The mixture was extracted with cyclohexane/CH_2_Cl_2_ 9:1 (4 × 10 mL), and the combined organic layer was washed with water (10 mL), dried over MgSO_4_, and concentrated under reduced pressure. The residue was purified by silica gel column chromatography.

## Supporting Information

File 1 General information, characterization data, and copies of NMR spectra.
