# Supplementary material for: Modular synthesis of 2-furyl carbinols from 3-benzyldimethylsilylfurfural platforms relying on oxygen-assisted C–Si bond functionalization
Source: Beilstein J Org Chem. 2022 Sep 16;18:1256–63. doi: 10.3762/bjoc.18.131 (PMC9490068; doi:10.3762/bjoc.18.131)
Supplement: File 1 — General information, characterization data, and copies of NMR spectra. [file Beilstein_J_Org_Chem-18-1256-s001.pdf]

BEILSTEIN JOURNAL OF ORGANIC CHEMISTRY

Supporting Information

for

Modular synthesis of 2-furyl carbinols from 3-benzyldimethylsilylfurfural platforms relying on oxygen-assisted C–Si bond functionalization

Sebastien Curpanen, Per Reichert, Gabriele Lupidi, Giovanni Poli, Julie Oble and Alejandro Perez-Luna

Beilstein J. Org. Chem. **2022,** *18,* 1256–1263. doi:10.3762/bjoc.18.131

General information, characterization data, and copies of NMR spectra

License and Terms: This is a supporting information file under the terms of the Creative Commons Attribution License (https://creativecommons.org/licenses/by/4.0). Please note that the reuse, redistribution and reproduction in particular requires that the author(s) and source are credited and that individual graphics may be subject to special legal provisions.
The license is subject to the *Beilstein Journal of Organic Chemistry* terms and conditions: (https://www.beilstein-journals.org/bjoc/terms)

Table of contents

I.	General information	S2
II.	Preparation of C3-silylated 2-furylcarbinols **3a–c**, **4c**, **5c**, **6c**, and **7c**	S3
III.	Rationalization of the formation of compounds **12** and **13** from **11**	S5
IV.	C3–$SiMe_2Bn$ bond functionalization through TBAF-promoted siloxane formation and cross-coupling	S6
	a. TBAF-promoted cyclic siloxane **16** formation (NMR monitoring)	S6
	b. Pd/Cu-catalyzed arylation of C3–$SiMe_2Bn$-appended 2-furylcarbinol **4c**	S7
	c. Copper-catalyzed allylation and alkylation of C3–$SiMe_2Bn$-appended 2-furylcarbinols	S8
V.	^{1}H and ^{13}C NMR spectra of new compounds	S12
VI.	References	S28

I. General information

All reactions were carried out under an argon atmosphere by standard syringe and septa techniques. Glassware was flame-dried under vacuum or taken directly from the oven (100 °C) and allowed to cool under vacuum prior to every use. Reagents and solvents were purchased from commercial sources and generally used as received. CH_2Cl_2 and Et_2O were dried on the MBraun purification system MB SPS-800. THF from the MB SPS-800 system was distilled over sodium and benzophenone under nitrogen flow prior to utilization. Acetonitrile and DMSO were distilled over calcium chloride and calcium hydride, respectively, before each use. DMF, 99.8%, extra dry over molecular sieves, AcroSeal®, was purchased from ACROS.

NMR spectra (^1H, ^{13}C, ^{19}F, and ^{31}P) were recorded on a Bruker AM 300 MHz or on a Bruker AVANCE 400 MHz spectrometer. NMR experiments were carried out at room temperature in $CDCl_3$. Chemical shift is given in parts per million (ppm) using the $CDCl_3$, and residual nondeuterated signals as reference (δ ^1H = 7.26 ppm; δ ^{13}C = 77.06 ppm). The terms m, s, d, t, q, and br s represent multiplet, singlet, doublet, triplet, quartet, and broad singlet, respectively. Coupling constants (J) are given in Hertz (Hz). For previously unknown compounds, a combination of ^{13}C, DEPT, and 2D NMR experiments (COSY, HSQC, and HMBC) were used.

IR spectra were recorded with a Tensor 27 (ATR Diamond) Bruker spectrophotometer. IR spectra were reported as characteristic bands (cm^{-1}). High-resolution mass spectra (ESIMS or APCIMS) were acquired using an LTQ-Orbitrap XL (Thermo Fisher Scientific, Courtaboeuf, France) operated in positive ionization mode. TLC analyses were performed on Merck 60 F_{254} silica gel and revealed with either an ultraviolet lamp (λ = 254 nm) or a specific staining reagent (potassium permanganate, *p*-anisaldehyde, etc.). Purifications by flash column chromatography were performed using silica gel Merck Geduran® SI 60 (40–63 µm).

The C3-silylated furfurals **1a**–**1c** and **2c** were prepared following our procedure reported in Reference [1].

II. Preparation of C3-silylated 2-furylcarbinols 3a–c, 4c, 5c, 6c, and 7c

General procedure for the addition of *n*-BuLi to C3-silylated furfurals (GP1). In a flame-dried round-bottom flask under argon was placed the appropriate C3-silylated furfural and dissolved in freshly distilled THF (0.3 M). The solution was cooled to −78 °C and then *n*-BuLi (solution in hexane, 1.2 equiv) was added dropwise. The reaction mixture was allowed to stir at −78 °C for 30 min and then quenched with aq sat. NH_4Cl/NH_3 2:1 solution. Et_2O was added and the aqueous layer was extracted three times. The combined organics were washed with brine, dried over $MgSO_4$, filtered, concentrated under reduced pressure and purified by silica gel column chromatography.

General procedure for the addition of Grignard reagents to C3-silylated furfurals (GP2). In a flame-dried round-bottom flask under argon was placed the appropriate C3-silylated furfural and dissolved in freshly distilled THF (0.2 M solution). The solution was cooled to 0 °C and then the Grignard reagent (solution in Et_2O, 1.3 equiv) was added dropwise (the rate of addition was equal or lower than 0.125 mL/min). The mixture was allowed to stir at 0 °C for 1 h, then allowed to reach room temperature. Upon consumption of the starting material, the reaction mixture was quenched with sat. NH_4Cl. CH_2Cl_2 was added and the aqueous layer was extracted three times (CH_2Cl_2). The combined organics were dried over Na_2SO_4, filtered, concentrated under reduced pressure and purified by silica gel column chromatography.

3a

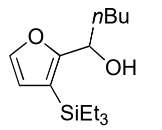

Chemical Formula: $C_{15}H_{28}O_2Si$
Molecular Weight: 268,47

1-(3-(Triethylsilyl)furan-2-yl)pentan-1-ol

Prepared according to GP1 from C3-silylated aldehyde **1a** (367 mg, 1.75 mmol). Purification by silica gel column chromatography (pentane/Et_2O 99:1 to 85:15) yielded 461 mg of **3a** (98% yield) as a yellow liquid. **^{1}H NMR** (400 MHz, $CDCl_3$) δ 7.42 (d, *J* = 1.7 Hz, 1H), 6.28 (d, *J* = 1.7 Hz, 1H), 4.62 (t, *J* = 7.1 Hz, 1H), 1.99–1.78 (m, 2H), 1.71 (s, 1H), 1.42–1.11 (m, 4H), 1.01-0.93 (m, 9H), 0.89 (t, *J* = 7.2 Hz, 3H), 0.82–0.68 (m, 6H). **^{13}C NMR** (101 MHz, $CDCl_3$) δ 160.9, 141.7, 115.2, 111.9, 68.5, 36.1, 28.2, 22.6, 14.1, 7.5, 4.3. **HRMS**: *m/z* calculated for $C_{15}H_{28}O_2SiNa$ $[M+Na]^+$ 291.1751, found 291.1751. **IR**: ν (cm^{-1}) 3350, 2955, 2875, 1756, 1678, 1416, 1127, 1016, 724.

3b

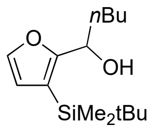

Chemical Formula: $C_{15}H_{28}O_2Si$
Molecular Weight: 268,47

1-(3-(*tert*-Butyldimethylsilyl)furan-2-yl)pentan-1-ol

Prepared according to GP1 from C3-silylated aldehyde **1b** (209 mg, 1.00 mmol). Purification by silica gel column chromatography (pentane/Et_2O 99:1 to 90:10), yielded 176 mg of **3b** (66% yield) as a yellow oil. **^{1}H NMR** (400 MHz, $CDCl_3$) δ 7.41 (d, *J* = 1.7 Hz, 1H), 6.29 (d, *J* = 1.8 Hz, 1H), 4.65 (dd, *J* = 7.7, 6.4 Hz, 1H), 2.00–1.78 (m, 2H), 1.34 (m, 4H), 0.89 (s, 12H 18H in the spectra), 0.25 (s, 3H), 0.22 (s, 3H). **^{13}C NMR** (101 MHz, $CDCl_3$) δ 161.0, 141.4, 115.5, 112.5, 68.3, 36.1, 28.2, 26.5, 22.6, 17.1, 14.1, –4.4, –4.7. **HRMS**: *m/z* calculated for $C_{15}H_{28}O_2SiNa$ $[M+Na]^+$ 291.1751, found 291.1751. **IR**: ν (cm^{-1}) 3401, 2955, 2929, 2857, 1662, 1510, 1464, 1390, 1250, 1133, 832.

3c

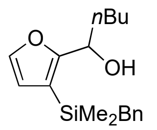

Chemical Formula: $C_{18}H_{26}O_2Si$
Molecular Weight: 302,48

1-(3-(Benzyldimethylsilyl)furan-2-yl)pentan-1-ol

Prepared according to GP1 from C3-silylated aldehyde **1c** (256 mg, 1.05 mmol). Purification by silica gel column chromatography (pentane/Et_2O 99:1 to 90:10), yielded 198 mg of **3c** (63% yield) as a yellow oil. **^{1}H NMR** (400 MHz, $CDCl_3$) δ 7.41 (d, *J* = 1.8 Hz, 1H), 7.24–7.15 (m, 2H), 7.12–7.05 (m, 1H), 6.98–6.91 (m, 2H), 6.27 (d, *J* = 1.7 Hz, 1H), 4.39 (t, *J* = 7.0 Hz, 1H), 2.29–2.22 (m, 2H), 1.89–1.66 (m, 2H), 1.38 (s, 1H (*OH*)), 1.34–1.23 (m, 3H), 1.13–1.10 (m, 1H), 0.88 (t, *J* = 7.1 Hz, 3H), 0.28 (s, 3H), 0.25 (s, 3H). **^{13}C NMR** (101 MHz, $CDCl_3$) δ 161.2, 141.6, 139.7, 128.4, 128.2, 124.4, 114.5, 112.8, 68.2, 35.6, 28.0, 26.9, 22.5, 14.0, –2.06, –2.10. **HRMS**: *m/z* calculated for $C_{18}H_{26}O_2SiNa$ $[M+Na]^+$ 325.1595, found 325.1594. **IR**: ν (cm^{-1}) 3367, 2929, 2861, 1684, 1575, 1494, 1453, 1377, 1251, 1186, 1109, 1033, 964, 890, 824, 795, 763, 699, 630, 558, 479, 413.

4c

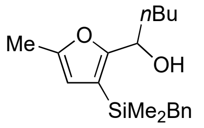

Chemical Formula: $C_{19}H_{28}O_2Si$
Molecular Weight: 316,51

1-(3-(Benzyldimethylsilyl)-5-methylfuran-2-yl)pentan-1-ol

Prepared according to GP1 from C3-silylated aldehyde **2c** (1.121 g, 4.33 mmol). Purification by silica gel column chromatography (pentane/Et_2O 99:1 to 90:10), yielded 1.03 g of **4c** (75% yield) as a yellow oil. **^{1}H NMR** (400 MHz, $CDCl_3$) *δ* 7.20 (t, *J* = 7.6 Hz, 2H), 7.08 (t, *J* = 7.3 Hz, 1H), 6.95 (d, *J* = 7.4 Hz, 2H), 5.84 (s, 1H), 4.35–4.26 (m, 1H), 2.28 (s, 3H), 2.23 (s, 2H), 1.86–1.67 (m, 2H), 1.36–1.22 (m, 3H+1H (*OH*)), 1.17–1.07 (m, 1H), 0.88 (t, *J* = 7.2 Hz, 3H), 0.23 (s, 3H), 0.21 (s, 3H). **^{13}C NMR** (101 MHz, $CDCl_3$) *δ* 159.7, 151.5, 139.9, 128.42, 128.32, 124.4, 119.6, 110.5, 68.6, 35.8, 28.2, 27.0, 22.6, 14.1, 13.4, –2.0. **HRMS**: *m/z* calculated for $C_{19}H_{28}O_2SiNa$ $[M+Na]^+$: 339.1751, found 339.1752**.** **IR**: ν (cm^{-1}) 3409, 3024, 2956, 1678*,* 1250, 994, 699, 479.

5c

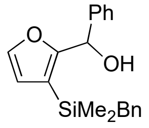

Chemical Formula: $C_{20}H_{22}O_2Si$
Molecular Weight: 322,47

(3-(Benzyldimethylsilyl)furan-2-yl)(phenyl)methanol

Prepared according to GP2 from C3-silylated aldehyde **1c** (244 mg, 1.00 mmol) and PhMgBr (2.7 M in Et_2O, 0.482 mL, 1.30 mmol). Purification by silica gel column chromatography (cyclohexane/ethyl acetate 5:1 to 1:1) yielded 268 mg of **5c** (83% yield) as a yellow oil. **^{1}H NMR** (400 MHz, $CDCl_3$) δ 7.34 (d, *J* = 1.7 Hz, 1H), 7.30–7.17 (m, 5H), 7.14 (t, J = 7.6 Hz, 2H), 7.03 (t, *J* = 7.3 Hz, 1H), 6.89 (d, *J* = 8.0 Hz, 2H), 6.23 (d, *J* = 1.7 Hz, 1H), 5.53 (d, *J* = 5.0 Hz, 1H), 2.25 (d(AB system), *J* = 13.7 Hz, 1H), 2.21 (d(AB system), *J* = 13.7 Hz, 1H), 1.87 (d, *J* = 5.1 Hz, 1H (*OH*)), 0.24 (s, 3H), 0.23 (s, 3H). **^{13}C NMR** (101 MHz, $CDCl_3$) δ 159.6, 142.3, 141.1, 139.6, 128.4 (2C), 128.3, 127.7, 126.4, 124.4, 114.7, 113.8, 70.0, 26.9, –2.0, –2.1. **HRMS**: *m/z* calculated for $C_{20}H_{22}O_2SiH$ $[M+H]^+$: 323.1462, found 323.1465. **IR**: ν (cm^{-1}) 3377, 3025, 2956, 1599, 1493, 1451, 1250, 1206, 1154, 1129, 1017, 895. 820, 792, 751, 696, 629, 557, 479, 418.

6c

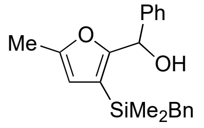

Chemical Formula: $C_{21}H_{24}O_2Si$
Molecular Weight: 336,50

(3-(Benzyldimethylsilyl)-5-methylfuran -2-yl)(phenyl)methanol

Prepared according to GP2 from C3-silylated aldehyde **2c** (78 mg, 0.30 mmol) and PhMgBr (2.7 M in Et_2O, 0.148 mL, 0.40 mmol). Purification by silica gel column chromatography (cyclohexane/ethyl acetate 5:1 to 1:1) yielded 72 mg of **6c** (71% yield) as a light-yellow oil. **^{1}H NMR** (400 MHz, $CDCl_3$) δ 7.27–7.25 (m, 4H), 7.25–7.12 (m, 3H), 7.06–7.00 (m, 1H), 6.93–6.88 (m, 2H), 5.82 (q, *J* = 1.0 Hz, 1H), 5.47 (d, *J* = 5.1 Hz, 1H), 2.22 (d, *J* = 2.6 Hz, 2H), 2.18 (d, *J* = 1.0 Hz, 3H), 1.91 (d, *J* = 5.1 Hz, 1H (*OH*)), 0.22 (s, 3H), 0.21 (s, 3H). **^{13}C NMR** (101 MHz, $CDCl_3$) δ 158.0, 152.2, 141.4, 139.7, 128.4, 128.3, 128.26, 127.5, 126.4, 124.4, 115.0, 110.6, 70.1, 26.9, 13.3, –2.0, –2.1. **HRMS** *m/z* calculated for $C_{21}H_{24}O_2SiH$ $[M+H]^+$ 337.1618, found 337.1620. **IR**: ν (cm^{-1}) 3377, 3025, 2956, 1599, 1493, 1250, 895, 751, 557, 479.

7c

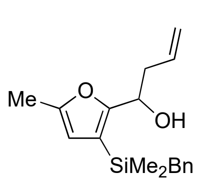

Chemical Formula: $C_{18}H_{24}O_2Si$
Molecular Weight: 300,47

1-(3-(Benzyldimethylsilyl)furan-2-yl)but-3-en-1-ol

Prepared according to GP2 from C3-silylated aldehyde **2c** (258 mg, 1.0 mmol) and allylmagnesium bromide (0.7 M in Et_2O, 4.28 mL, 1.30 mmol). Purification by silica gel column chromatography (cyclohexane/ethyl acetate 80:20) afforded 210 mg of **7c** (70% yield) as a light-yellow oil. **^{1}H NMR** (400 MHz, $CDCl_3$) δ 7.20 (t, *J* = 7.4 Hz, 2H), 7.08 (t, *J* = 7.4 Hz, 1H), 6.95 (d, *J* = 7.4 Hz, 2H), 5.85 (d, *J* = 1.0 Hz, 1H), 5.73–5.62 (m, 1H), 5.13–5.04 (m, 2H), 4.36 (ddd, *J* = 7.8, 6.0, 4.5 Hz, 1H), 2.61–2.42 (m, 2H), 2.28 (d, *J* = 1.0 Hz, 3H), 2.24 (s, 2H), 1.55 (d, *J* = 4.5 Hz, 1H (*OH*)), 0.23 (s, 3H), 0.21 (s, 3H). **^{13}C NMR** (101 MHz, $CDCl_3$) δ 158.7, 151.6, 139.8, 134.3, 128.4, 128.2, 124.3, 118.1, 114.3, 110.5, 67.5, 40.5, 26.9, 13.3, –2.08, –2.12. **HRMS** *m/z* calculated for $C_{18}H_{24}O_2SiH$ $[M+H]^+$ 301.1618, found 301.1618. **IR**: ν (cm^{-1}) 3424, 3080, 2954, 1642, 1493, 1337, 1250, 994, 828, 558, 479.

III. Rationalization of the formation of compounds 12 and 13 from 11.

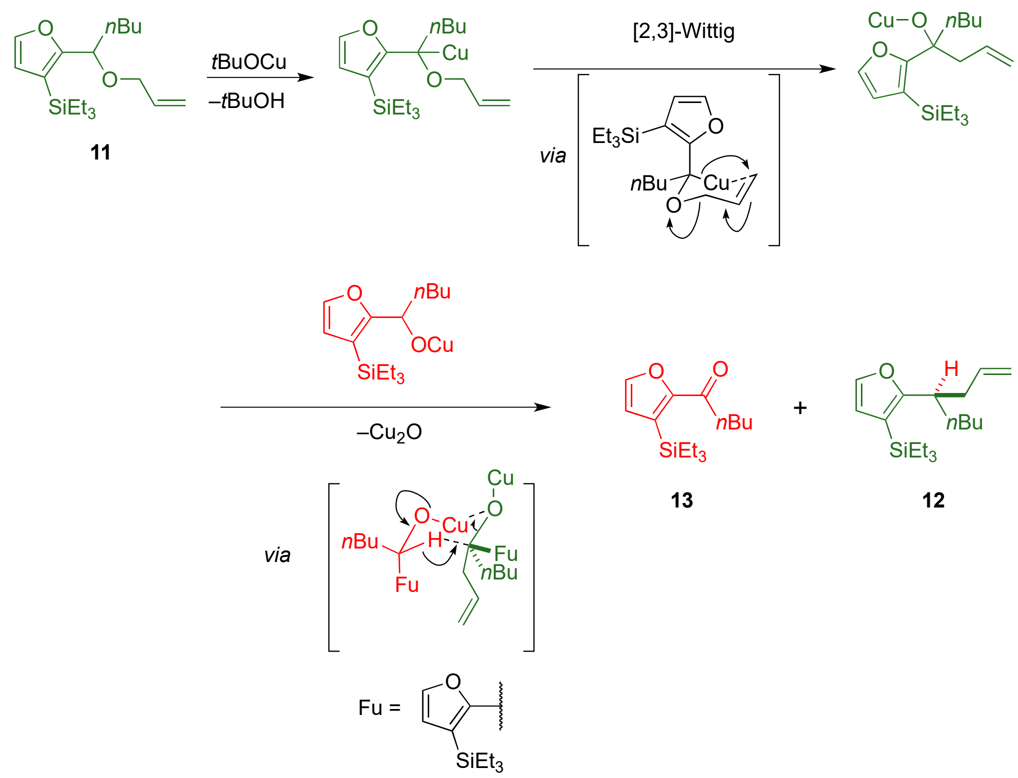

IV. C3–SiMe$_2$Bn bond functionalization through TBAF-promoted siloxane formation and cross-coupling

a. TBAF-promoted cyclic siloxane 16 formation (NMR monitoring)

NMR monitoring of the formation of the reaction between **4c** and TBAF·3H_2O (formation of **16**):

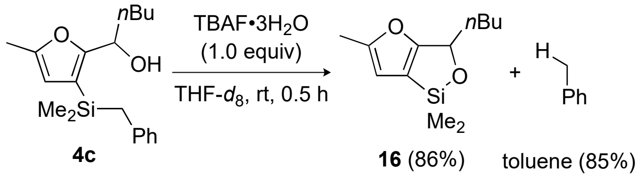

In an NMR tube, to a THF-d_8 (0.5 mL) solution of **4c** (32 mg, 0.10 mmol) was added TBAF·3H_2O (35 mg, 0.11 mmol) and the mixture was stirred under argon overnight. 3,4,5-Methoxybenzaldehyde (11,3 mg, 0.058 mmol) was added as internal standard. A ^{1}H NMR spectrum was recorded and analyzed: it evidenced the formation of 89% of siloxane **16** and 85% of toluene.

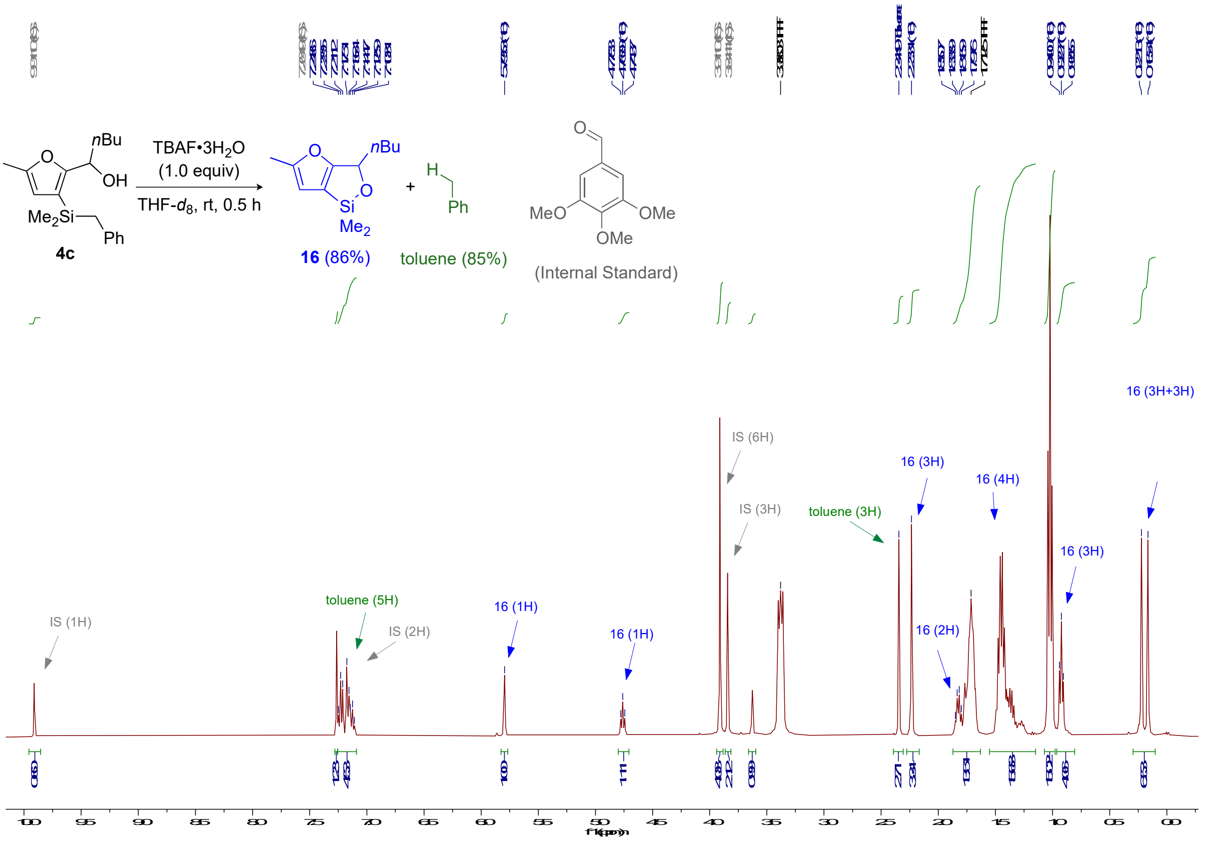

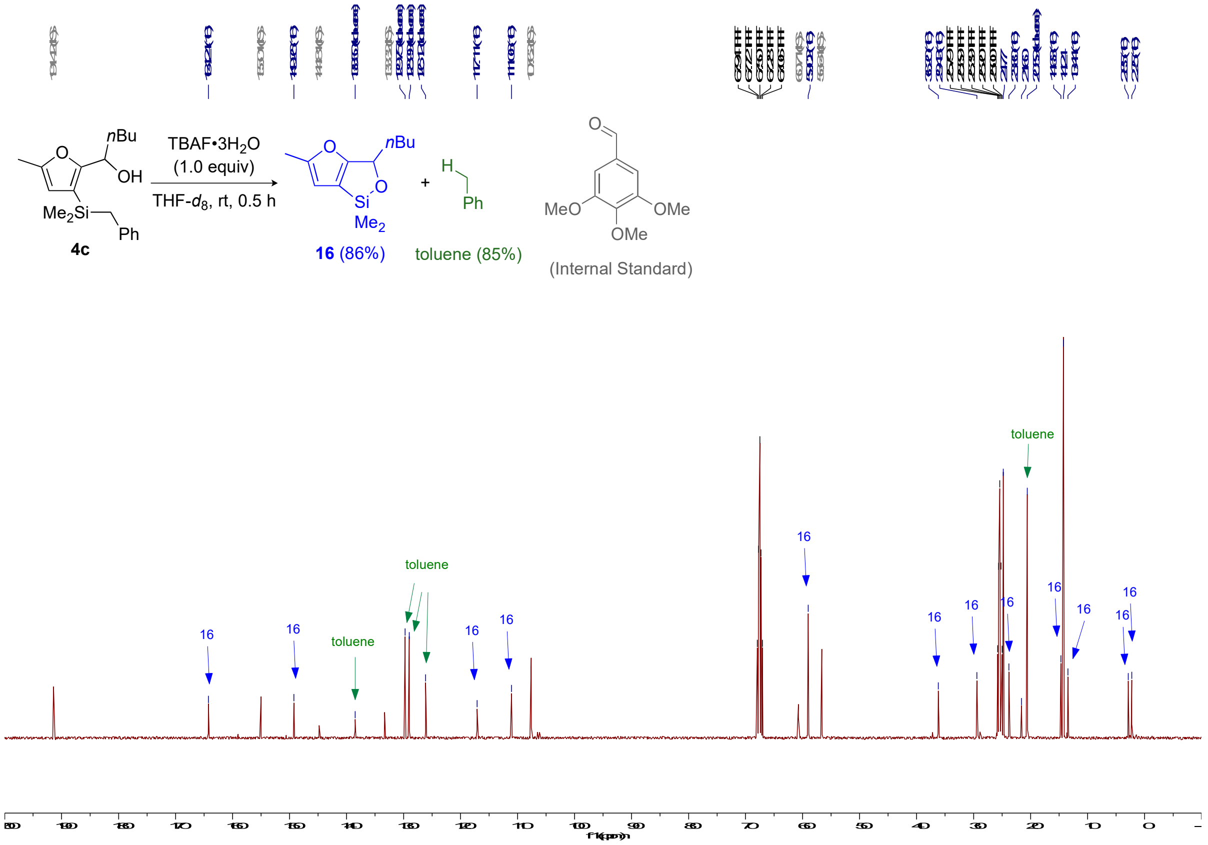

Product **16** could not be purified by silica gel chromatography. It was nevertheless characterized conveniently by ^{1}H and ^{13}C NMR spectroscopy.

16

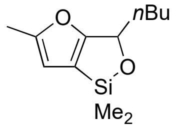

Chemical Formula: $C_{12}H_{20}O_2Si$
Molecular Weight: 224,37

3-Butyl-1,1,5-trimethyl-1,3-dihydrofuro[3,2-c][1,2]oxasilole

^{1}H NMR (400 MHz, THF-d_8) δ 5.80 (s, 1H), 4.77 (t, J = 7.0 Hz, 1H), 2.24 (s, 3H), 1.83 (q, J = 7.2 Hz, 2H), 1.41–1.16 (m, 4H), 0.93 (t, J = 7.0 Hz, 3H), 0.23 (s, 3H), 0.17 (s, 3H). **^{13}C NMR** (101 MHz, THF-d_8) δ 164.2, 149.3, 117.1, 111.1, 59.0, 36.2, 29.4, 23.8, 14.7, 13.4, 2.9, 2.3.

b. Pd/Cu-Catalyzed arylation of C3–SiMe$_2$Bn-appended 2-furylcarbinol 4c

General procedure (GP 3): A flame-dried Schlenk tube was charged with CuI (20 mol %) and $Pd_2(dba)_3$ (2.5 mol %) and heated gently under vacuum by means of a heat gun. In another Schlenk tube, the appropriate iodoarene (1.5 equiv) and **4c** (1 equiv) were dissolved in freshly distilled THF (0.3 M). The solution was degassed by 3 cycles of freeze–pump–thaw and degassed anhydrous TBAF (1 M in THF, 2.2 equiv) was added. The mixture was allowed to stir for 10 min at 0 °C and transferred via cannula to the Schlenk tube containing the catalyst. The resulting mixture was stirred for 2 h at rt, then filtered through a short pad of silica gel and concentrated. The residue was purified by silica gel column chromatography.

18

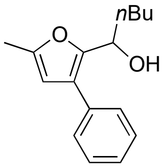

Chemical Formula: $C_{16}H_{20}O_2$
Molecular Weight: 244,33

1-(5-Methyl-3-phenylfuran-2-yl)pentan-1-ol

Prepared according to GP3 from **4c** (60 mg, 0.19 mmol) and iodobenzene (32 µL, 0.28 mmol). Purification by silica gel column chromatography (cyclohexane/ethyl acetate 90:10) yielded 33 mg of **18** (70% yield) as a yellow oil. **^{1}H NMR** (400 MHz, $CDCl_3$) δ 7.31-7.27 (m, 4H), 7.19 (ddt, J = 9.0, 6.5, 3.4 Hz, 1H), 6.01 (s, 1H), 4.65 (t, J = 7.2 Hz, 1H), 2.23 (s, 3H), 1.95–1.71 (m, 3H), 1.35–1.04 (m, 4H), 0.78 (t, J = 7.0 Hz, 3H). **^{13}C NMR** (101 MHz, $CDCl_3$) δ 151.3, 149.5, 133.8, 128.7, 128.3, 127.0, 124.4, 107.7, 66.5, 35.5, 28.2, 22.6, 14.1, 13.7. **HRMS** *m/z* calculated for $C_{16}H_{20}O_2H$ [M+H]$^+$: 245.1535, found 245.1536. **IR**: ν (cm^{-1}) 2955, 2857, 1575, 1456, 1289, 1246, 1108, 1015.

19

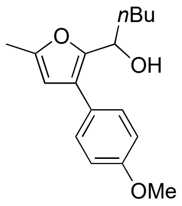

Chemical Formula: $C_{17}H_{22}O_3$
Molecular Weight: 274,35

1-(3-(4-Methoxyphenyl)-5-methylfuran-2-yl)pentan-1-ol

Prepared according to GP3 from **4c** (37 mg, 0.12 mmol) and 4-iodoanisole (41 mL, 0.18 mmol). Purification by silica gel column chromatography (cyclohexane/ethyl acetate 90:10) yielded 18 mg of **19** (57% yield) as a yellow liquid. **^{1}H NMR** (400 MHz, $CDCl_3$) δ 7.31 (d, *J* = 8.3 Hz, 2H), 6.93 (d, *J* = 8.4 Hz, 2H), 6.07 (s, 1H), 4.72 (t, *J* = 7.3 Hz, 1H), 3.83 (s, 3H), 2.31 (s, 3H), 2.06–1.77 (m, 2H), 1.83 (s, 1H (*OH)*), 1.46–1.19 (m, 4H), 0.88 (t, *J* = 7.0 Hz, 3H). **^{13}C NMR** (101 MHz, $CDCl_3$) δ 158.8, 151.1, 149.0, 129.3, 126.3, 124.0, 114.2, 107.8, 66.5, 55.4, 35.5, 28.2, 22.6, 14.1, 13.7. **HRMS** *m/z* calculated for $C_{17}H_{22}O_3Na$ $[M+Na]^+$: 297.1461, found 297.1460. **IR**: ν (cm^{-1}) 2955, 2857, 2138, 1575, 1456, 1289, 1108, 1015.

c. Copper-catalyzed allylation and alkylation of C3–$SiMe_2Bn$-appended 2-furylcarbinols

Reagents:

TBAF·(*t*-BuOH)$_4$

According to a reported procedure [2], a commercially available anhydrous THF solution of TBAF (1 M, 10 mL, 10 mmol) was concentrated. Then *t*-BuOH (30 mL) was added, followed by *n*-hexane (6 mL). The mixture was stirred for 30 min at 90 °C, cooled to room temperature, and concentrated to about half of the solution upon which the formation of a white crystalline solid was observed. The solid was filtered using a Büchner funnel and washed with *t*-BuOH rapidly. The solid was kept under vacuum for 15–20 min and recovered. 5.3 g of TBAF·(*t*-BuOH)$_4$ (95% yield) was obtained as white crystals. **^{1}H NMR** (400 MHz, $CDCl_3$) *δ* 3.49 ppm (t, *J* = 9.0 Hz, 8H), 1.67–1.71 (m, 8H), 1.44–1.48 (m, 8H), 1.27 (s, 36H), 1.01 (t, *J* = 9.0 Hz, 12H). **^{19}F NMR** (376 MHz, $CDCl_3$) *δ* 109.08. These data are in good agreement with those reported in literature.

CuI·PPh$_3$

According to a reported procedure [3], triphenylphosphine (161 mg, 0.525 mmol) was dissolved in acetonitrile (2.0 mL) at 35 °C and added to a solution of copper(I) iodide (100 mg, 0.525 mmol) in acetonitrile (10.0 mL) at the same temperature. After a few seconds the iodo(triphenylphosphine)copper complex started to precipitate. The mixture was stirred for 1 h. The solid was filtered using a Büchner funnel, washed with acetonitrile (20.0 mL), and vacuum-dried. 231 mg of CuI·PPh$_3$ (97% yield) were obtained as a white crystalline powder. **^{31}P-NMR** (161 MHz, $CDCl_3$) *δ* –49.46. These data are in good agreement with those reported in literature.

General procedure (GP 4): CuI·PPh$_3$ (20 or 120 mol %) was introduced in a flame-dried microwave vial which was then placed under argon atmosphere and sealed. In a Schlenk tube, the appropriate C3–$SiMe_2Bn$-substituted 2-furylcarbinol (0.3 mmol, 1 equiv) was dissolved in CH_2Cl_2 (1 mL), concentrated under reduced pressure and placed under argon. DMF (3.0 mL) was then added and the solution was degassed by doing 3 freeze–pump–thaw cycles. TBAF·(*t*-BuOH)$_4$ (402 mg, 0.72 mmol) was then added along with the correspondding electrophile (3 equiv). The mixture was stirred for 2–3 min at rt, then cannulated into the vial containing the copper complex. The mixture was stirred for 2 h at 30 °C, then quenched with aq KOH (6 M, 1 mL). The mixture was extracted with cyclohexane/CH_2Cl_2 9:1 (4 ×10 mL), the combined organics were washed with water (10 mL), dried over $MgSO_4$ and concentrated under reduced pressure. The residue was purified by silica gel column chromatography.

21

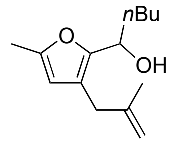

Chemical Formula: $C_{14}H_{22}O_2$
Molecular Weight: 222,32

1-(5-Methyl-3-(2-methylallyl)furan-2-yl)pentan-1-ol

Prepared according to GP4 from C3–$SiMe_2Bn$ 2-furylcarbinol **4c** (95 mg, 0.30 mmol) and methallyl chloride (88 μL, 0.90 mmol) using CuI•PPh_3 (27 mg, 0.060 mmol). The crude was analyzed by ^{1}H NMR and the product quantified using dimethyl sulfone as internal standard providing 84% NMR yield of **21** and about 1% of protodesilylated product **22**. The crude was purified by silica gel column chromatography (pentane/Et_2O, 90:10) to afford 52 mg of **21** (78% yield), as a pale-yellow oil. **^{1}H NMR** (400 MHz, $CDCl_3$) δ 5.78 (s, 1H), 4.73 (m, 2H), 4.61 (m, 1H), 3.05 (s, 2H), 2.24 (s, 3H), 1.95–1.77 (m, 2H), 1.81 (br. s, 1H (*OH*)), 1.70 (s, 3H), 1.39–1.15 (m, 4H), 0.88 (d, *J* = 7.2 Hz, 3H). **^{13}C NMR** (101 MHz, $CDCl_3$) δ 150.8, 150.0, 145.0, 119.8, 111.2, 108.4, 66.3, 35.5, 33.4, 28.1, 22.6, 22.4, 14.1, 13.7. **HRMS** *m/z* calculated for $C_{14}H_{22}O_2H$ $[M+H]^+$: 223.1693, found 223.1693. **IR**: ν (cm^{-1}) 3360, 3076, 1642, 1575, 1251, 993, 914, 416.

23

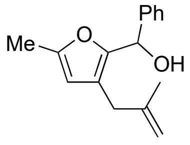

Chemical Formula: $C_{16}H_{18}O_2$
Molecular Weight: 242,31

(5-Methyl-3-(2-methylallyl)furan-2-yl)(phenyl)methanol

Prepared according to GP4 from C3–$SiMe_2Bn$-substituted 2-furylcarbinol **6c** (101 mg, 0.30 mmol) and methallyl chloride (88 μL, 0.90 mmol) and using CuI·PPh_3 (27 mg, 0.060 mmol). The crude was purified by silica gel column chromatography (pentane/Et_2O 90:10) to afford 40 mg of **23** (56% yield) as a yellow liquid. **^{1}H NMR** (400 MHz, $CDCl_3$) δ 7.25 (d, *J* = 7.4 Hz, 2H), 7.17 (t, *J* = 7.5 Hz, 2H), 7.13–7.07 (m, 1H), 5.64 (s, 2H), 4.62–4.51 (m, 2H), 2.87 (s, 2H), 2.17 (s, 1H (*OH*)), 2.04 (s, 3H), 1.51 (s, 3H). **^{13}C NMR** (101 MHz, $CDCl_3$) δ 151.4, 148.7, 144.7, 141.5, 128.3, 127.5, 126.4, 120.5, 111.4, 108.6, 68.1, 33.4, 22.3, 13.6. **HRMS** *m/z* calculated for $C_{16}H_{18}O_2H$ $[M+H]^+$: 243.1380, found 243.1380. **IR**: ν (cm^{-1}) 3391, 2995, 1600, 1493, 1145, 888, 732, 479.

24

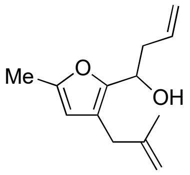

Chemical Formula: $C_{13}H_{18}O_2$
Molecular Weight: 206,28

1-(5-Methyl-3-(2-methylallyl)furan-2-yl)but-3-en-1-ol

Prepared according to GP4 from C3–$SiMe_2Bn$-appended 2-furylcarbinol **7c** (91 mg, 0.30 mmol) and methallyl chloride (88 μL, 0.90 mmol) using CuI·PPh_3 (163 mg, 0.36 mmol). The crude was purified by silica gel column chromatography (pentane/Et_2O 90:10) to afford 40 mg of **24** (65% yield) as a pale yellow oil. **^{1}H NMR** (400 MHz, $CDCl_3$) δ 5.86–5.70 (m, 2H), 5.24–5.06 (m, 2H), 4.76 (s, 1H), 4.70 (s, 1H), 4.69–4.62 (m, 1H), 3.06 (s, 2H), 2.72–2.50 (m, 2H), 2.25 (d, *J* = 1.1 Hz, 3H), 1.89 (d, *J* = 4.5 Hz, 1H (*OH*)), 1.70 (s, 3H). **^{13}C NMR** (101 MHz, $CDCl_3$) δ 151.0, 149.2, 144.9, 134.4, 120.1, 118.2, 111.3, 108.5, 65.3, 40.4, 33.4, 22.4, 13.7. **HRMS** *m/z* calculated for $C_{13}H_{18}O_2H$ $[M+H]^+$: 207.1380, found 207.1380. **IR**: ν (cm^{-1}) 3424, 2954, 1642, 1493, 1155, 915, 762, 699, 629, 558, 479.

25

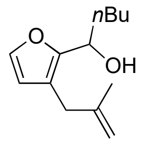

Chemical Formula: $C_{13}H_{20}O_2$
Molecular Weight: 208,30

+

25’

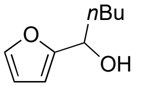

Chemical Formula: $C_9H_{14}O_2$
Molecular Weight: 154,21

1-(3-(2-Methylallyl)furan-2-yl)pentan-1-ol

Prepared according to GP4 from C3–$SiMe_2Bn$-appended 2-furylcarbinol **3c** (91 mg, 0.30 mmol) and methallyl chloride (88 μL, 0.90 mmol) using CuI·PPh_3 (27 mg, 0.060 mmol). The crude was purified by silica gel column chromatography (pentane/Et_2O 90:10) to afford 37 mg of a mixture of **25** (42% yield) and protodesilylated product **25’** [4] in a **25**/**25’** 64:36 ratio. Characterization of **25** was possible from this mixture: **^{1}H NMR** (400 MHz, $CDCl_3$) δ 7.30 (d, *J* = 1.8 Hz, 1H), 6.20 (d, *J* = 1.8 Hz, 1H), 4.78 (s, 1H), 4.70 (s, 1H), 4.66 (q, *J* = 4.4 Hz, 1H), 3.12 (s, 2H), 1.96–1.81 (m, 2H), 1.79 (d, *J* = 4.9 Hz, 1H), 1.71 (t, *J* = 1.1 Hz, 3H), 1.47–1.12 (m, 4H), 0.89 (dt, *J* = 9.3, 7.1 Hz, 3H). **^{13}C NMR** (101 MHz, $CDCl_3$) δ 151.7, 144.7, 141.1, 118.9, 112.4, 111.3, 66.1, 35.4, 33.2, 27.9, 22.5, 22.3, 14.1. **HRMS** *m/z* calculated for $C_{13}H_{20}O_2H$ $[M+H]^+$: 209.1536, found 209.1535.

26

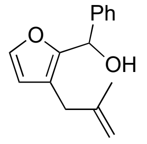

Chemical Formula: $C_{15}H_{16}O_2$
Molecular Weight: 228,29

+

26’

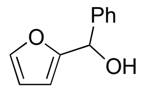

Chemical Formula: $C_{11}H_{10}O_2$
Molecular Weight: 174,20

(3-(2-Methylallyl)furan-2-yl)(phenyl)methanol

Prepared according to GP4 from C3–$SiMe_2Bn$-appended 2-furylcarbinol **5c** (97 mg, 0.30 mmol) and methallyl chloride (88 μL, 0.90 mmol) using CuI·PPh_3 (27 mg, 0.060 mmol). The crude was purified by silica gel column chromatography (pentane/Et_2O 90:10) to afford 30 mg of **26** (40% yield) as a yellow oil contaminated with 13% of protodesilylated product **26’**. **^{1}H NMR** (400 MHz, Chloroform-*d*) δ 7.37–7.18 (m, 6H), 6.15 (s, 1H), 5.79 (d, *J* = 4.3 Hz, 1H), 4.72 (s, 1H), 4.63 (s, 1H), 3.05 (s, 2H), 2.25 (d, *J* = 5.5 Hz, 1H (*OH*)), 1.62 (s, 3H). **^{13}C NMR** (101 MHz, $CDCl_3$) δ 150.6, 144.5, 141.7, 141.2, 128.4, 127.7, 126.4, 119.6, 112.6, 111.6, 68.2, 33.3, 22.3. **HRMS** *m/z* calculated for $C_{15}H_{16}O_2H$ $[M+H]^+$: 229.1223, found 229.1224. **IR**: ν (cm^{-1}) 3359, 2915, 1652, 1493, 1177, 891, 699, 418.

27

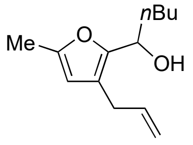

Chemical Formula: $C_{13}H_{20}O_2$
Molecular Weight: 208,30

+

27’

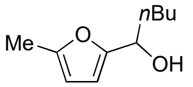

Chemical Formula: $C_{10}H_{16}O_2$
Molecular Weight: 168,23

1-(3-Allyl-5-methylfuran-2-yl)pentan-1-ol

Prepared according to GP4 from C3–$SiMe_2Bn$-appended 2-furylcarbinol (95 mg, 0.30 mmol) and allyl bromide (78 μL, 0.90 mmol) using CuI·PPh_3 (163 mg, 0.36 mmol). The crude was purified by silica gel column chromatography (pentane/Et_2O 90:10) to afford 43 mg of **27** (61% yield) as a pale yellow liquid contaminated with 14% of protodesilylated product **27’**. **^{1}H NMR** (300 MHz, $CDCl_3$) δ 5.97–5.82 (m, 1H), 5.84 (s, 1H), 5.12–4.90 (m, 2H), 4.62 (m, 1H), 3.12 (d, *J* = 6.3, 2H), 2.24 (s, 3H), 1.95–1.77 (m, 2H), 1.72 (brs, 1H (*OH*)), 1.40–1.15 (m, 4H), 0.89 (t, *J* = 7.1 Hz, 3H). **^{13}C NMR** (75 MHz, $CDCl_3$) δ 150.8, 149.4, 137.1, 119.7, 115.2, 108.0, 66.1, 35.4, 29.1, 28.0, 22.5, 14.0, 13.5. **HRMS** *m/z* calculated for $C_{13}H_{20}O_2H$ $[M+H]^+$: 209.1536, found 209.1536. **IR**: ν (cm^{-1}) 3356, 3079, 2956, 1640, 1574, 1036, 993, 785.

28

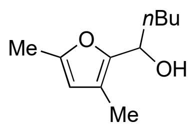

Chemical Formula: $C_{11}H_{18}O_2$
Molecular Weight: 182,26

1-(3,5-Dimethylfuran-2-yl)pentan-1-ol

Prepared according to GP4 from C3–$SiMe_2Bn$-appended 2-furylcarbinol **4c** (95 mg, 0.30 mmol) and iodomethane (56 μL , 0.90 mmol) using CuI·PPh_3 (27 mg, 0.06 mmol). The crude was purified by silica gel column chromatography (pentane/Et_2O 90:10) to afford 33 mg of **28** (61% yield) as a pale yellow oil. **^{1}H NMR** (400 MHz, $CDCl_3$) δ 5.76 (s, 1H), 4.65–4.57 (m, 1H), 2.22 (s, 3H), 1.97 (s, 3H), 1.95–1.76 (m, 2H), 1.71 (brs, 1H (*OH*)), 1.41–1.28 (m, 3H), 1.25–1.13 (m, 1H), 0.89 (t, *J* = 7.0 Hz, 3H). **^{13}C NMR** (101 MHz, $CDCl_3$) δ 150.6, 149.1, 117.1, 109.1, 66.1, 35.5, 28.0, 22.5, 14.1, 13.5, 9.7. **HRMS** *m/z* calculated for $C_{11}H_{18}O_2H$ [M+H]$^+$: 183.1380, found 183.1379. **IR**: ν (cm^{-1}) 3356, 2956, 1640, 1574, 1036, 993, 736, 698.

V. ^{1}H and ^{13}C NMR spectra of new compounds

1-(3-(triethylsilyl)furan-2-yl)pentan-1-ol (3a)

^{1}H NMR (400 MHz, $CDCl_3$)

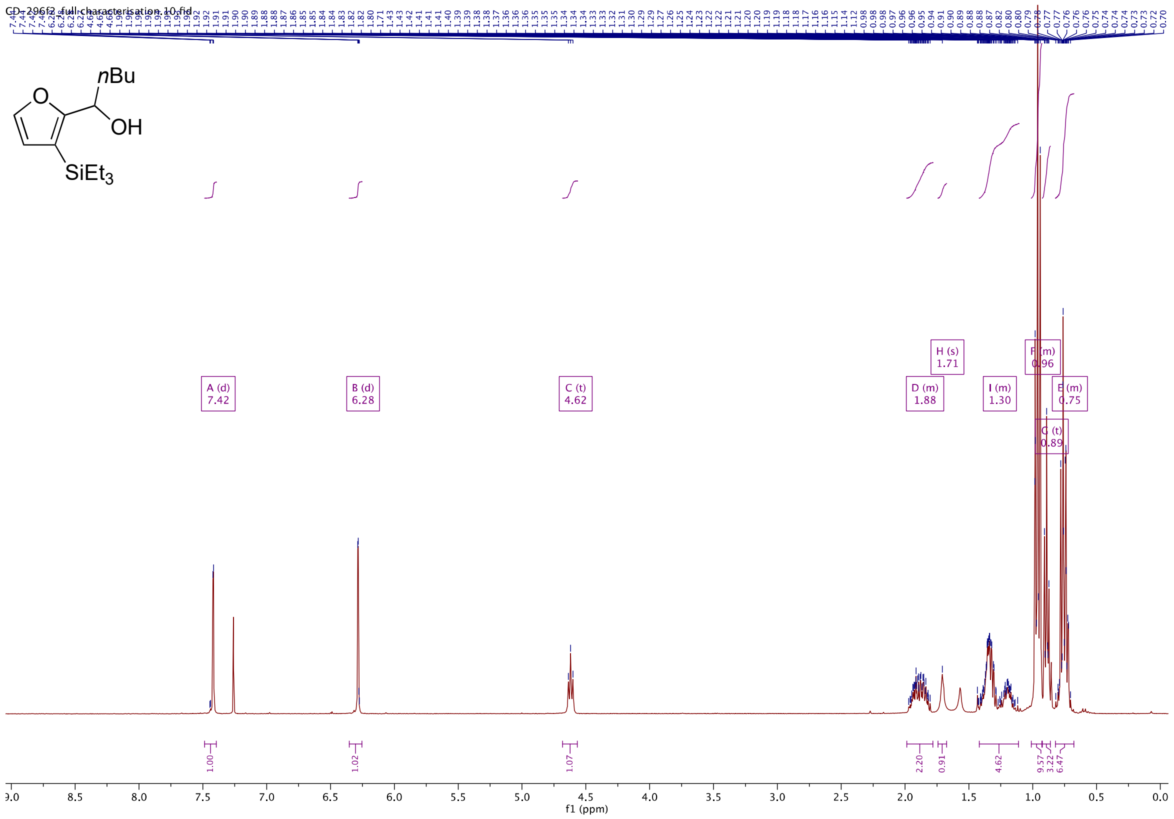

^{13}C Jmod NMR (100 MHz, $CDCl_3$)

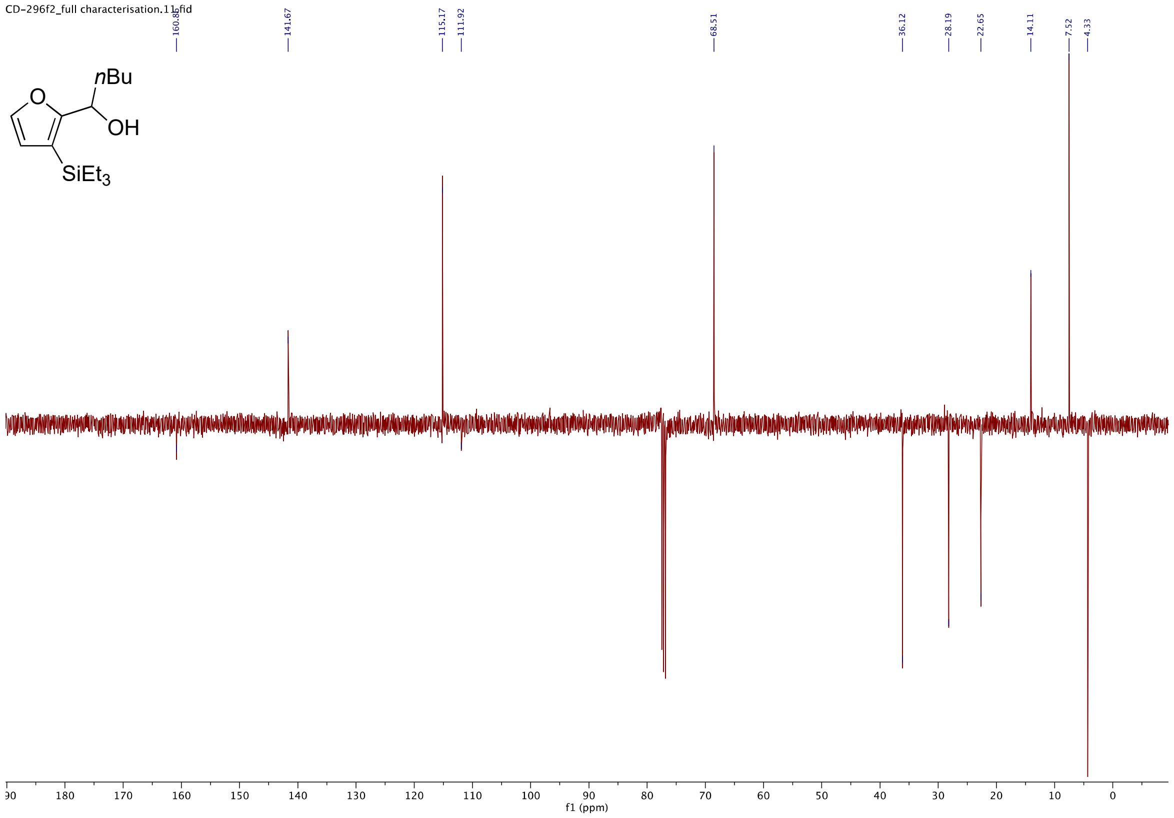

1-(3-(*t*-butyldimethylsilyl)furan-2-yl)pentan-1-ol (3b)

^{1}H NMR (400 MHz, $CDCl_3$)

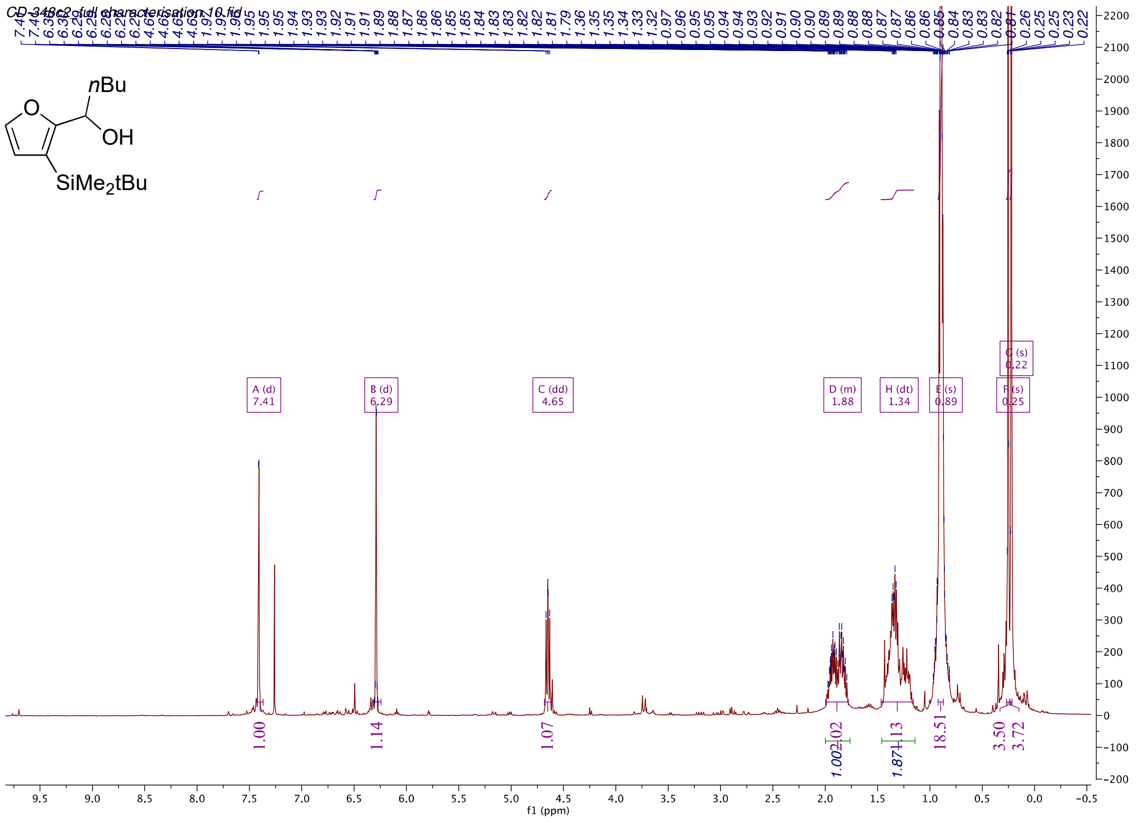

^{13}C Jmod NMR (100 MHz, $CDCl_3$)

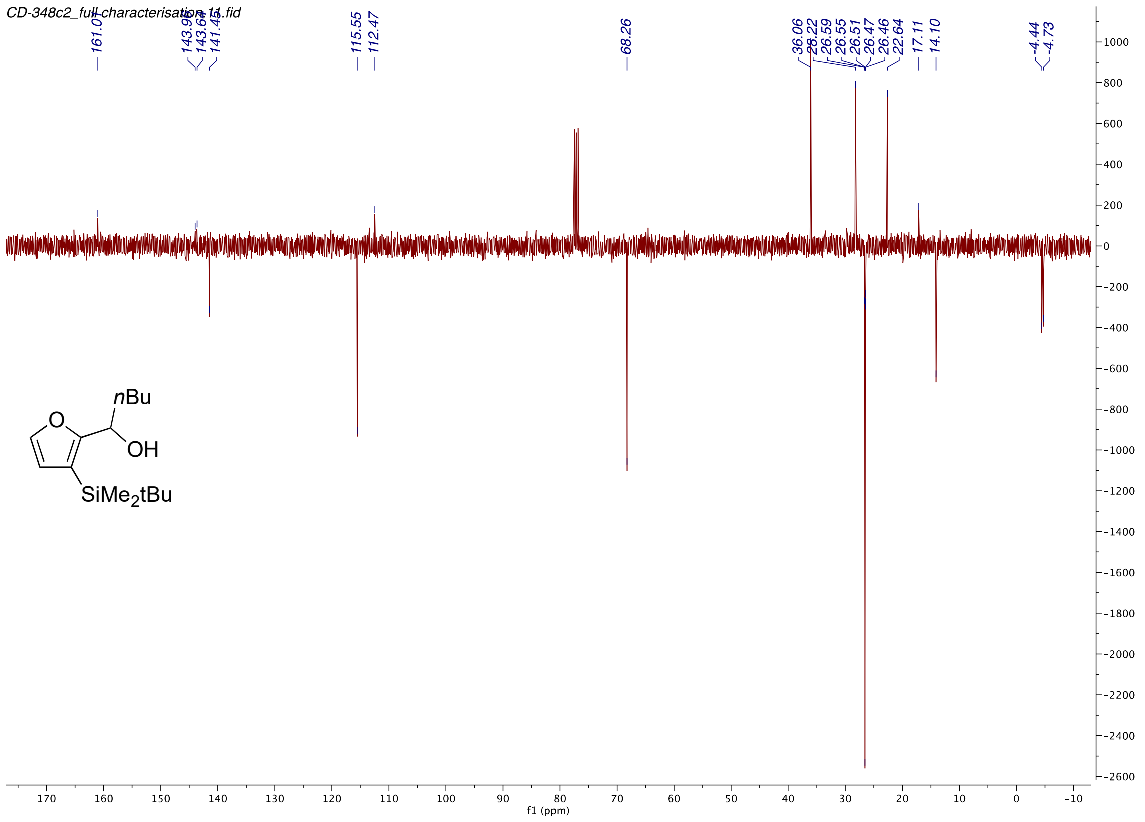

1-(3-(benzyldimethylsilyl)furan-2-yl)pentan-1-ol (3c)

^{1}H NMR (400 MHz, $CDCl_3$)

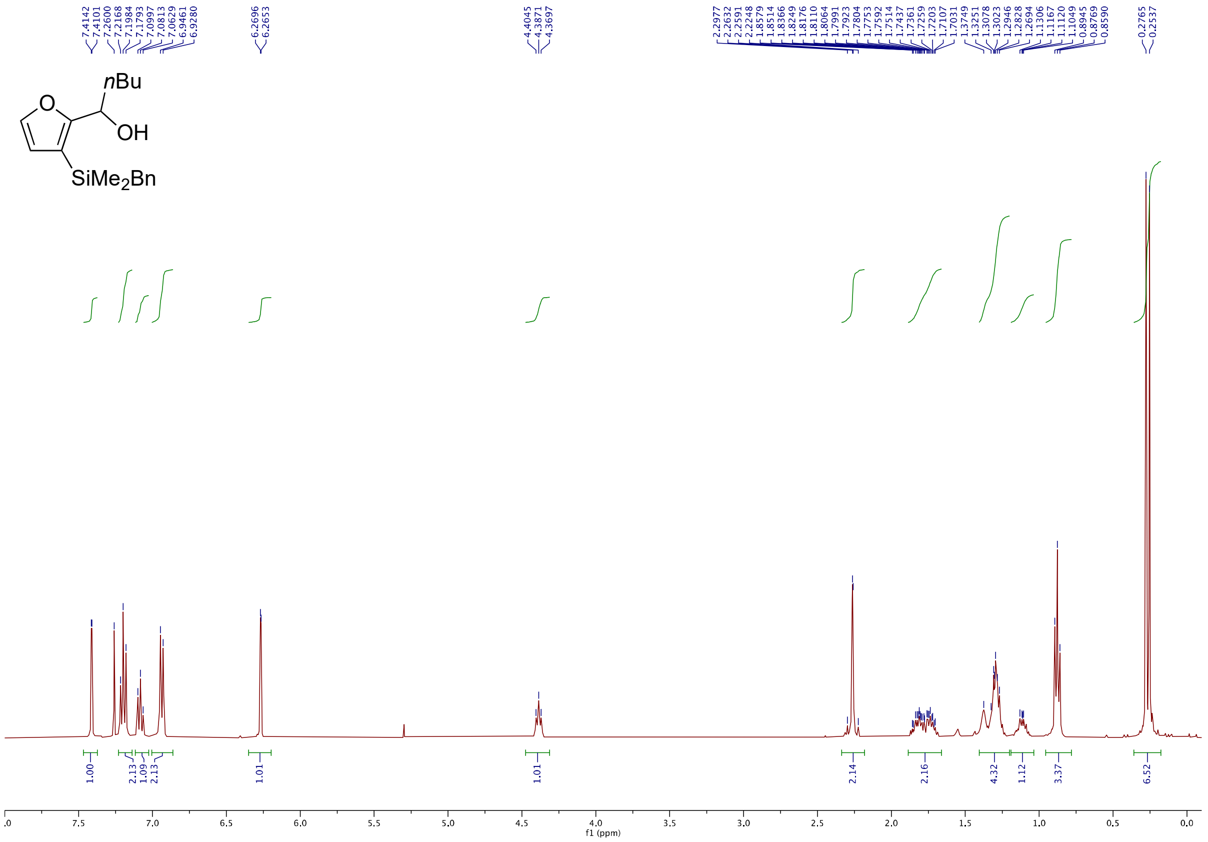

^{13}C NMR (100 MHz, $CDCl_3$)

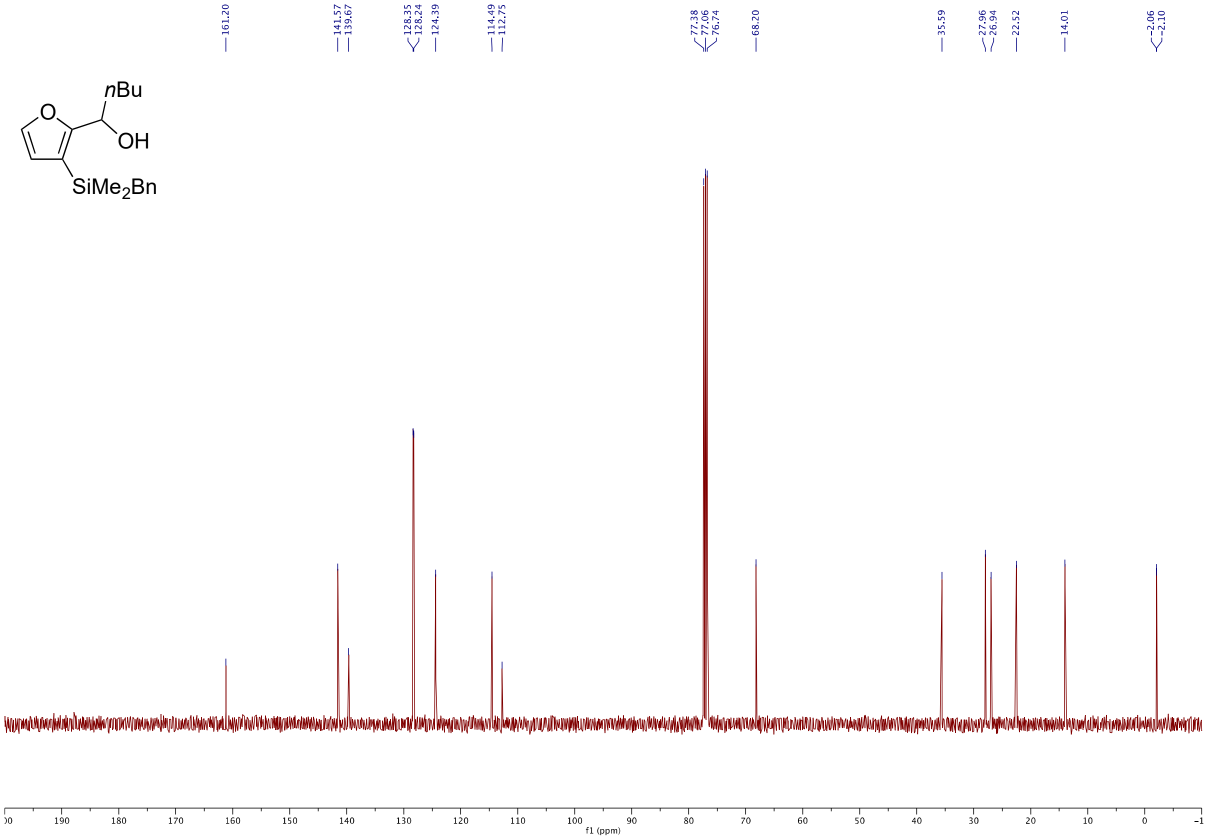

1-(3-(benzyldimethylsilyl)-5-methylfuran-2-yl)pentan-1-ol (4c)

^{1}H NMR (400 MHz, $CDCl_3$)

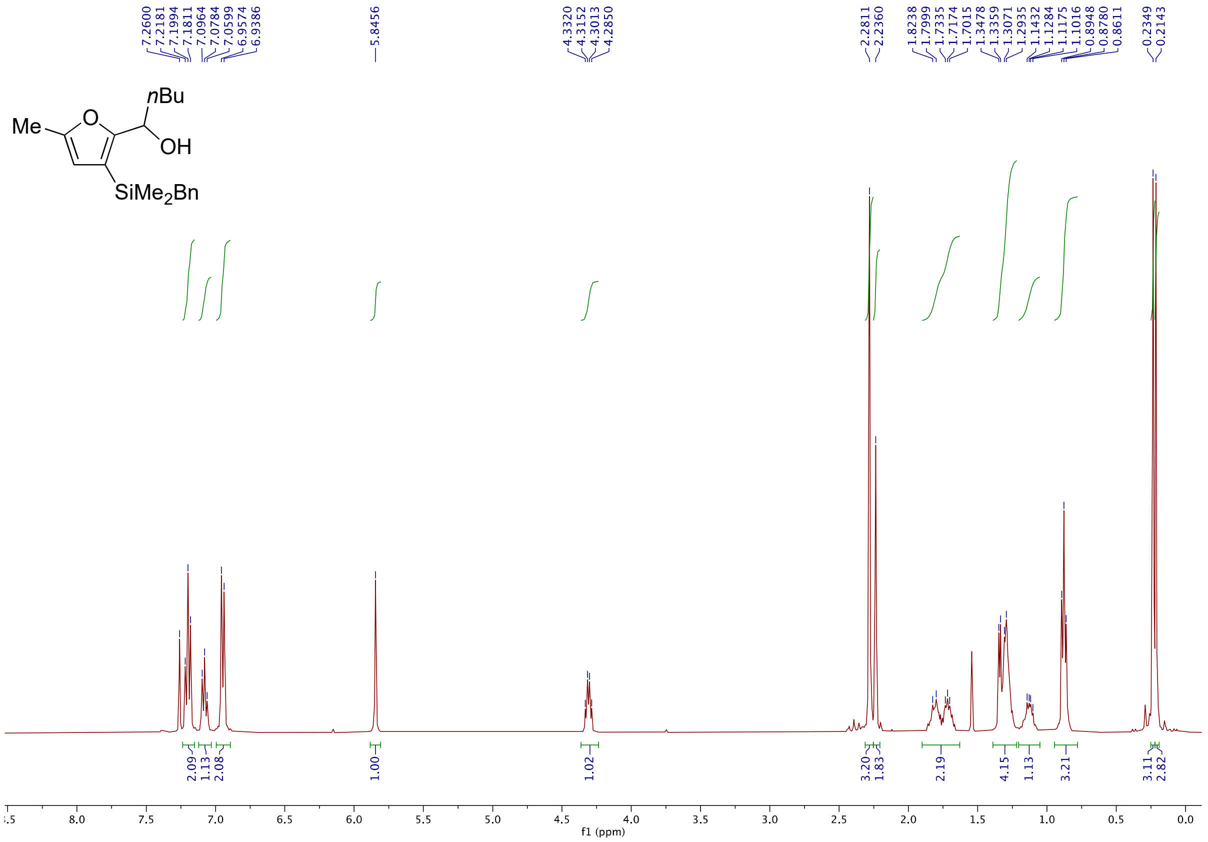

^{13}C Jmod NMR (100 MHz, $CDCl_3$)

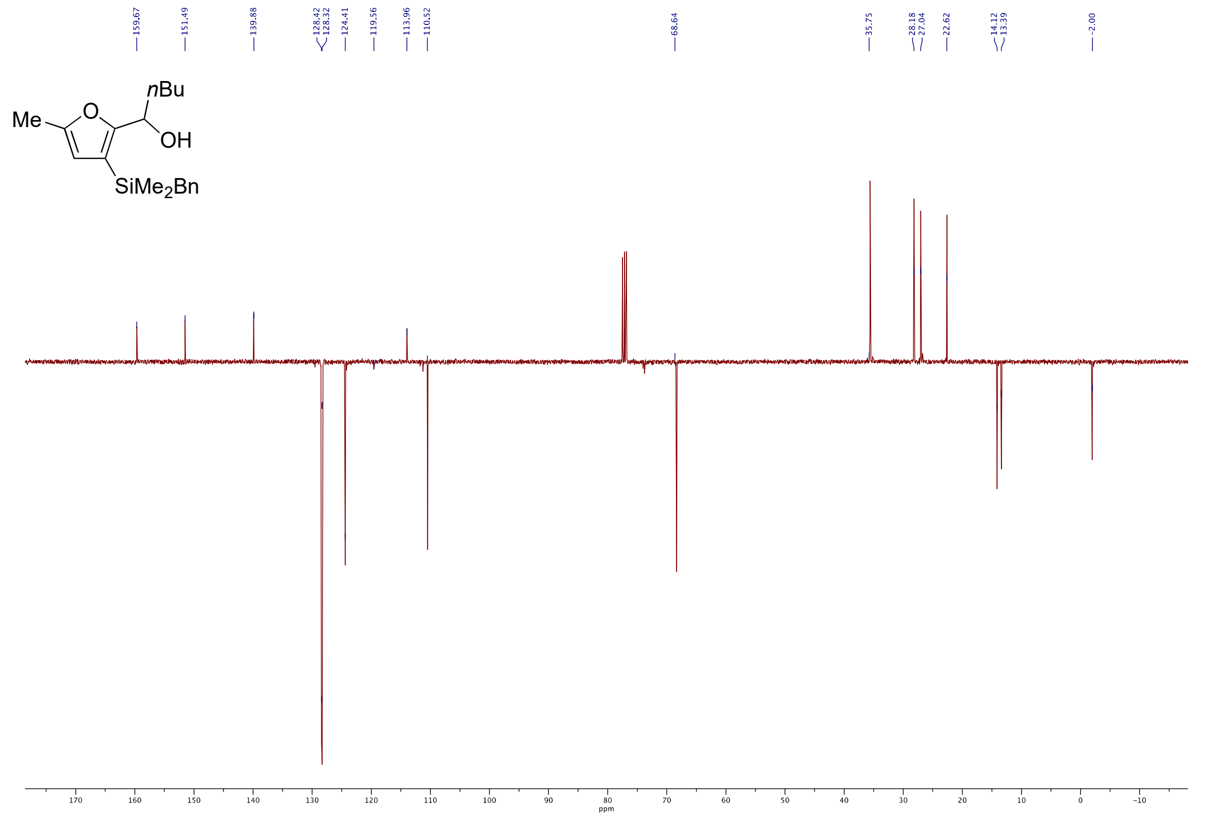

(3-(benzyldimethylsilyl)furan-2-yl)(phenyl)methanol (5c)

^{1}H NMR (400 MHz, $CDCl_3$)

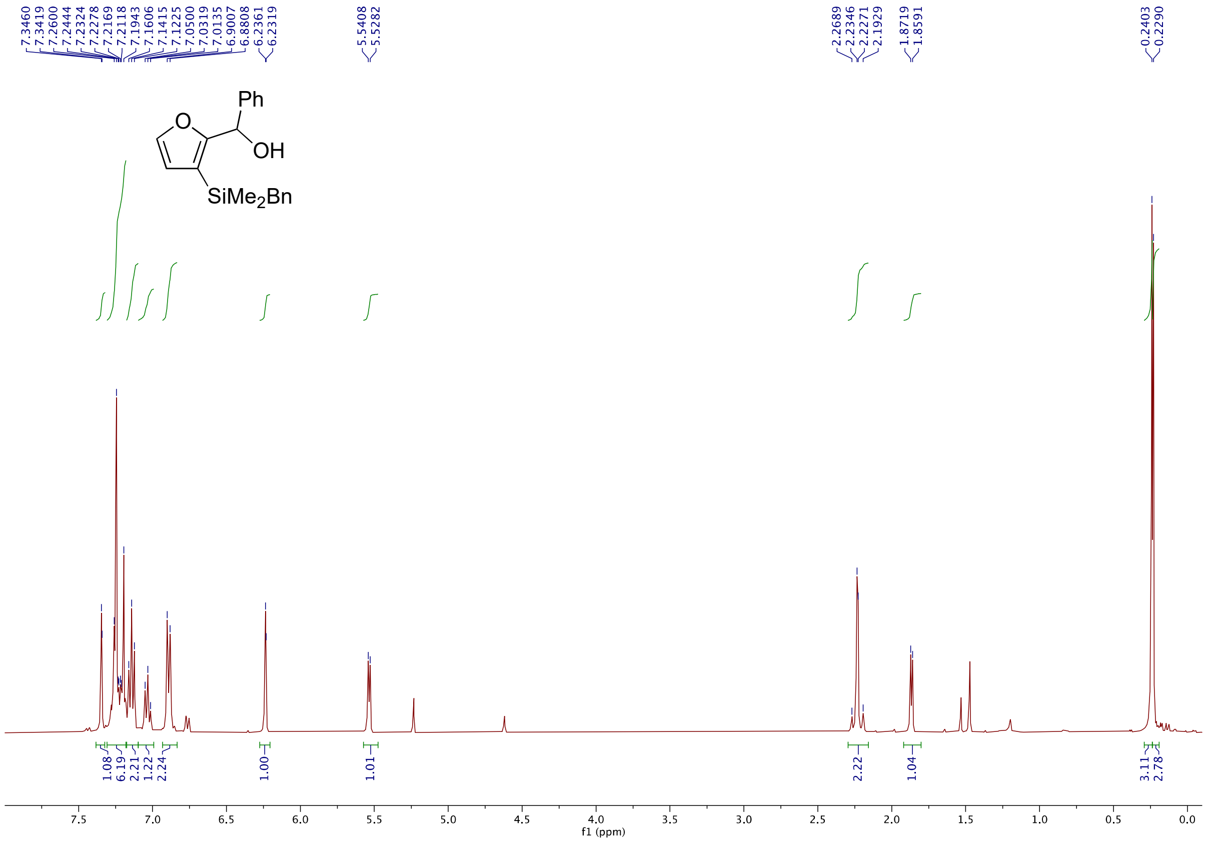

^{13}C Jmod NMR (100 MHz, $CDCl_3$)

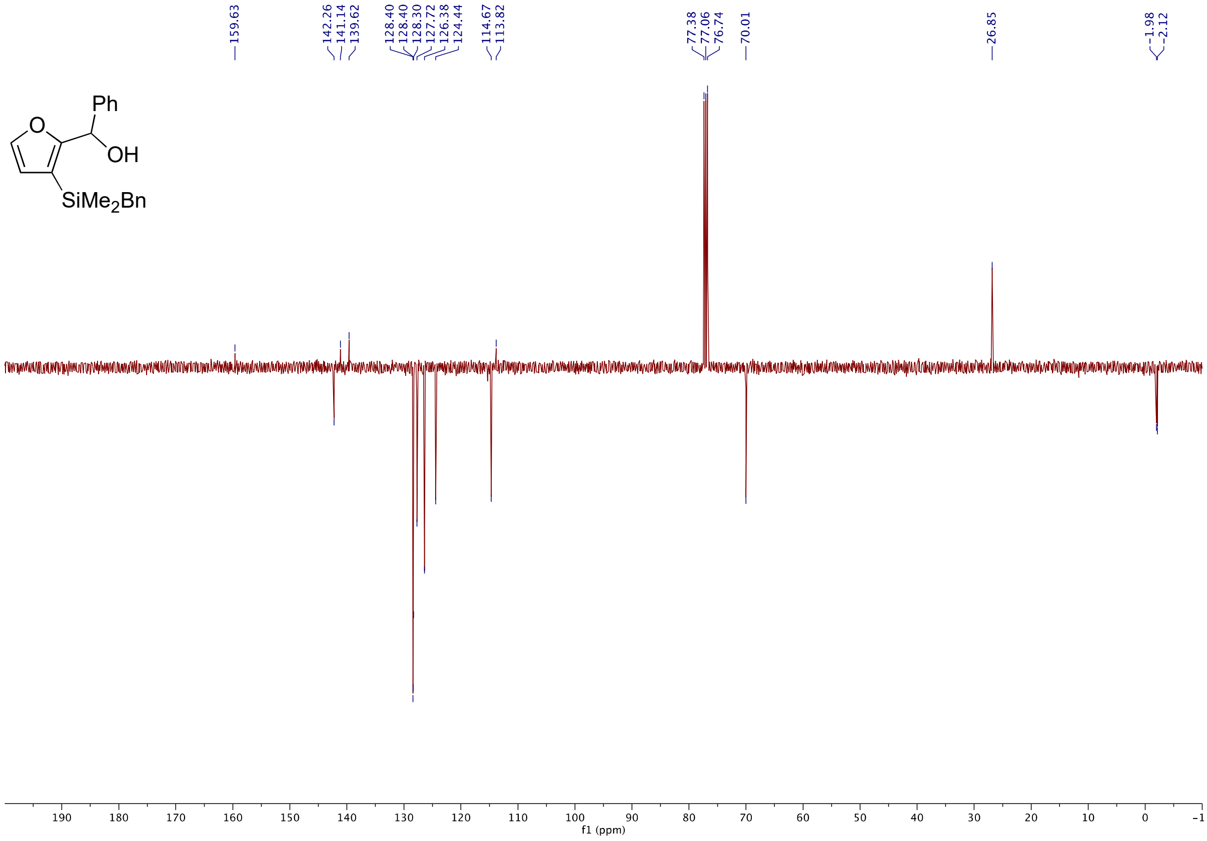

(3-(benzyldimethylsilyl)-5-methylfuran -2-yl)(phenyl)methanol (6c)

^{1}H NMR (400 MHz, $CDCl_3$)

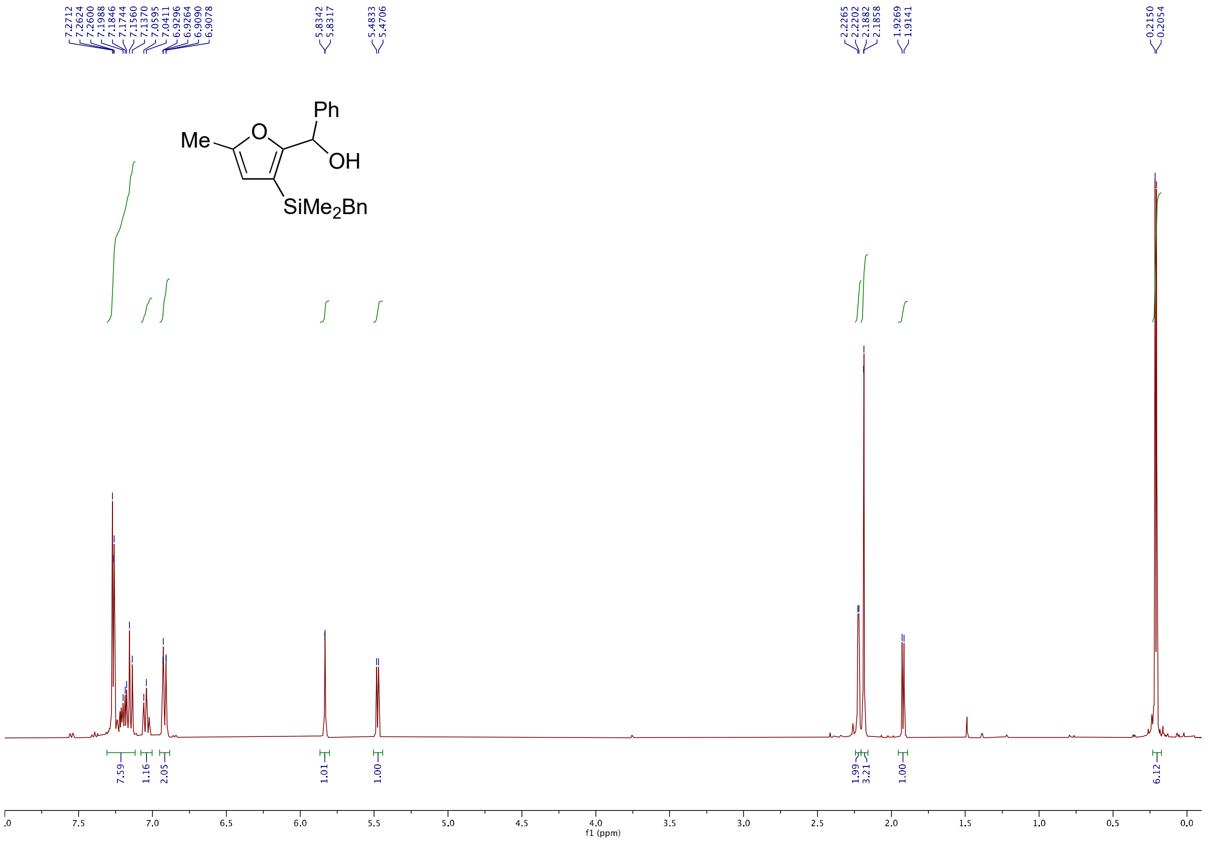

^{13}C Jmod NMR (100 MHz, $CDCl_3$)

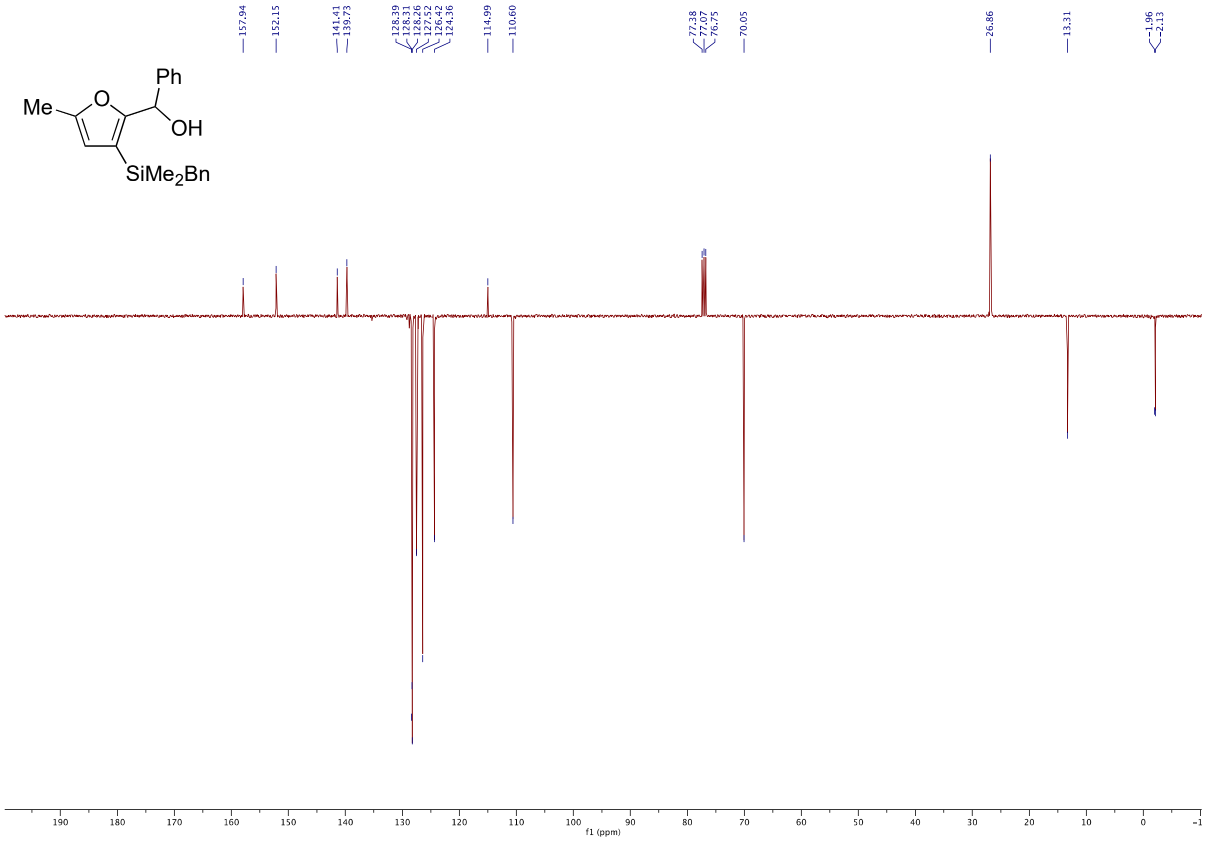

1-(3-(benzyldimethylsilyl)furan-2-yl)but-3-en-1-ol (7c)

^{1}H NMR (400 MHz, $CDCl_3$)

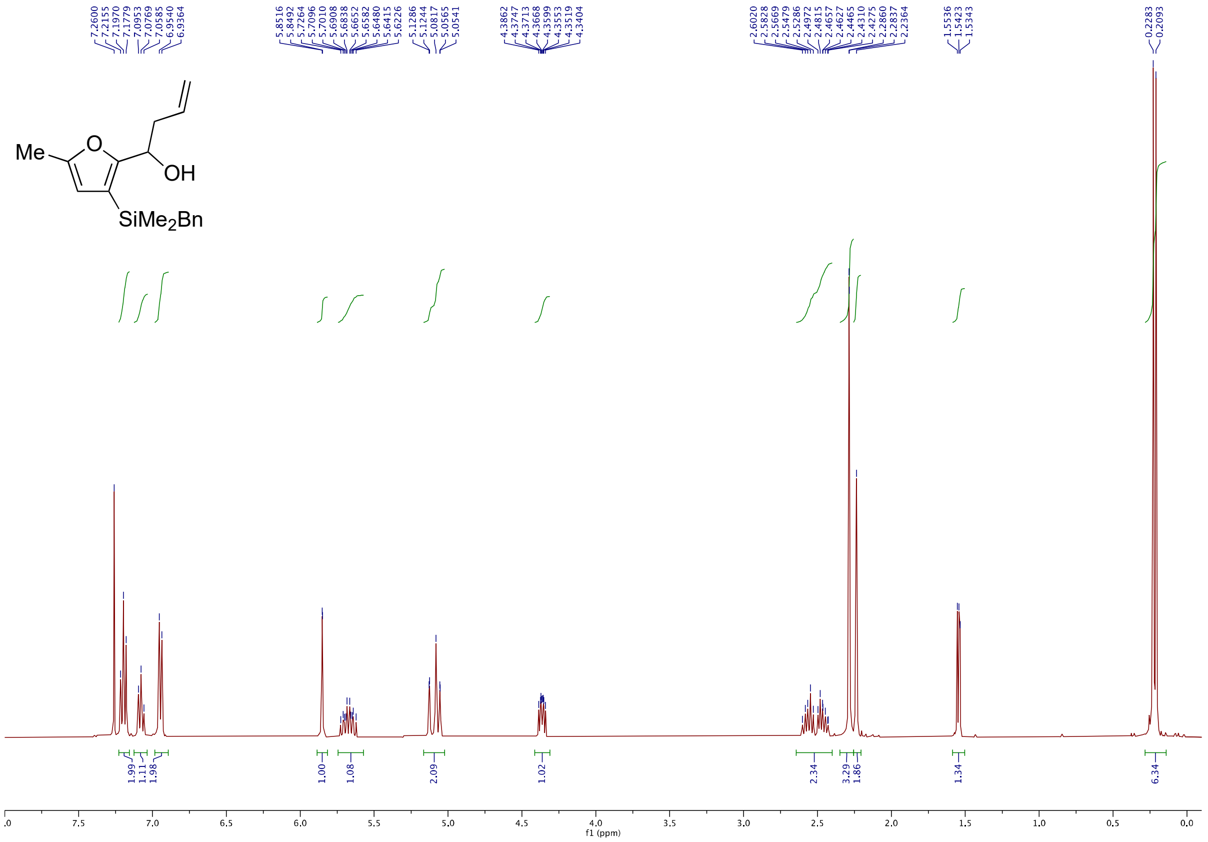

^{13}C Jmod NMR (100 MHz, $CDCl_3$)

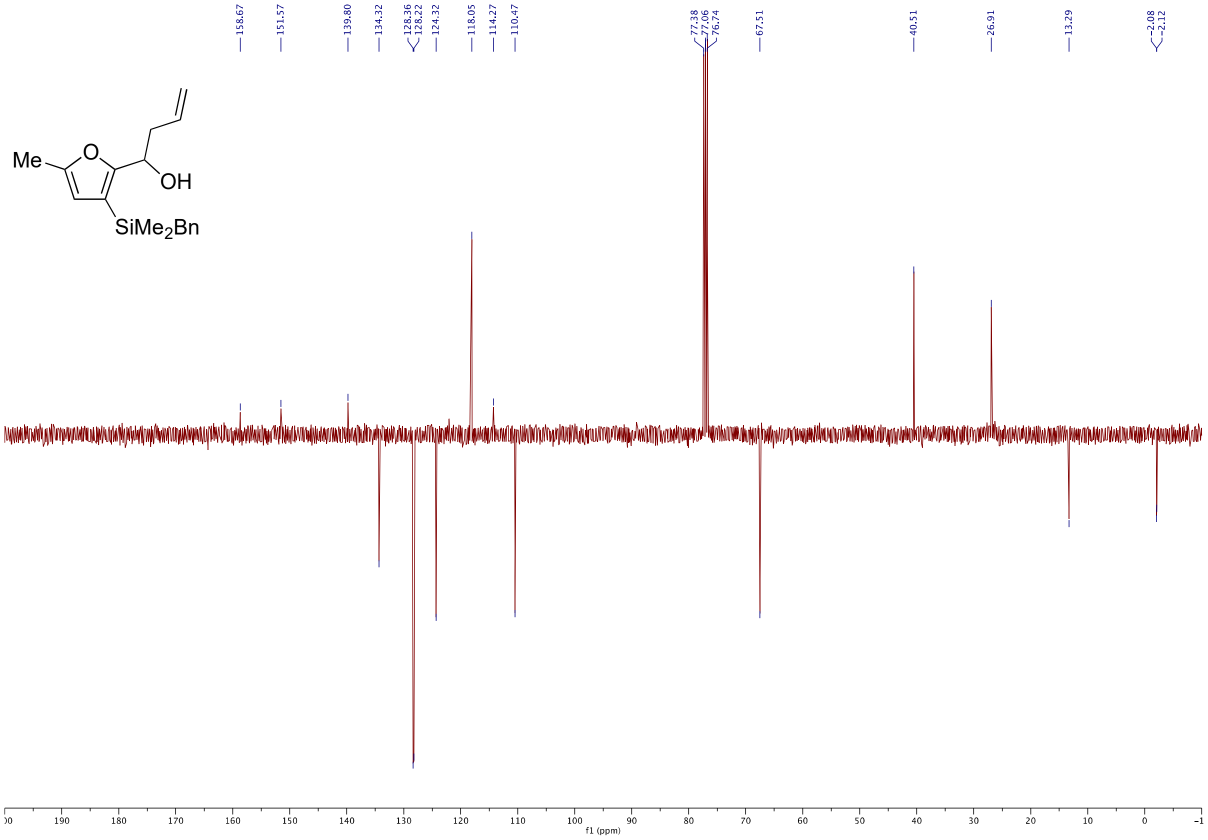

1-(5-methyl-3-phenylfuran-2-yl)pentan-1-ol (18)

^{1}H NMR (400 MHz, $CDCl_3$)

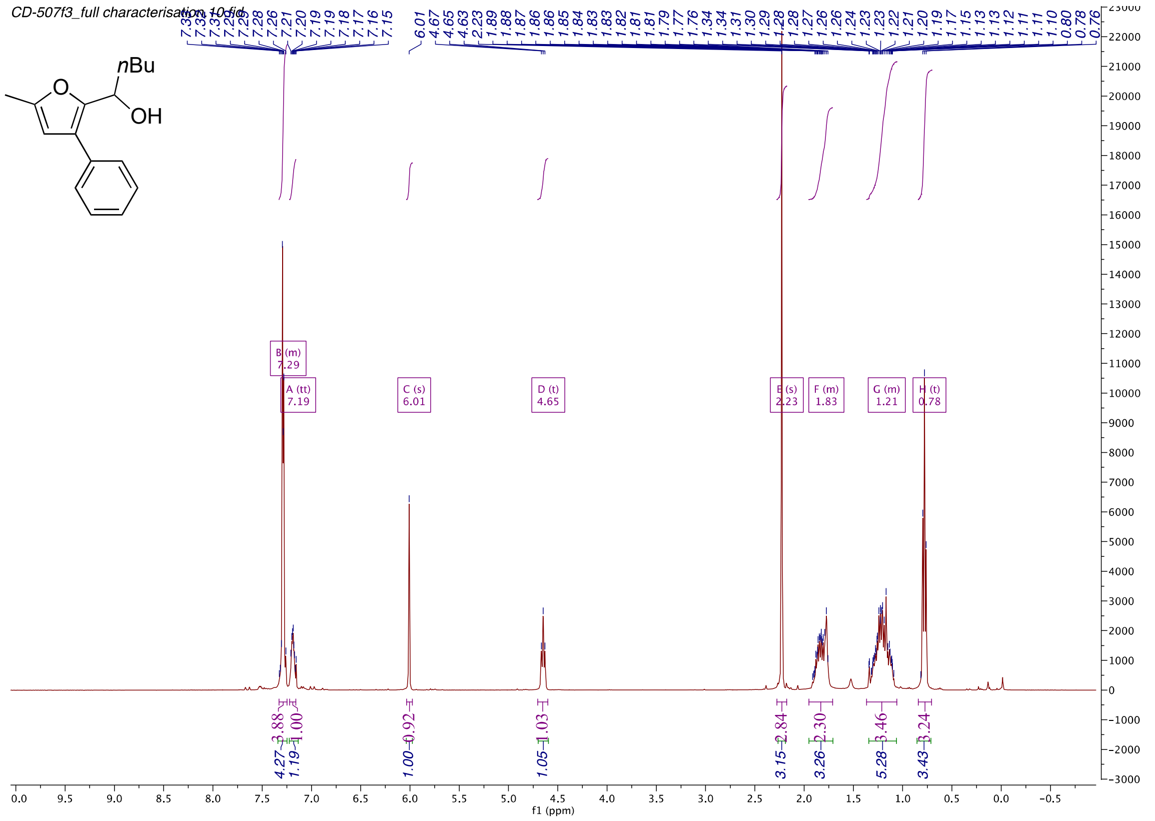

^{13}C Jmod NMR (100 MHz, $CDCl_3$)

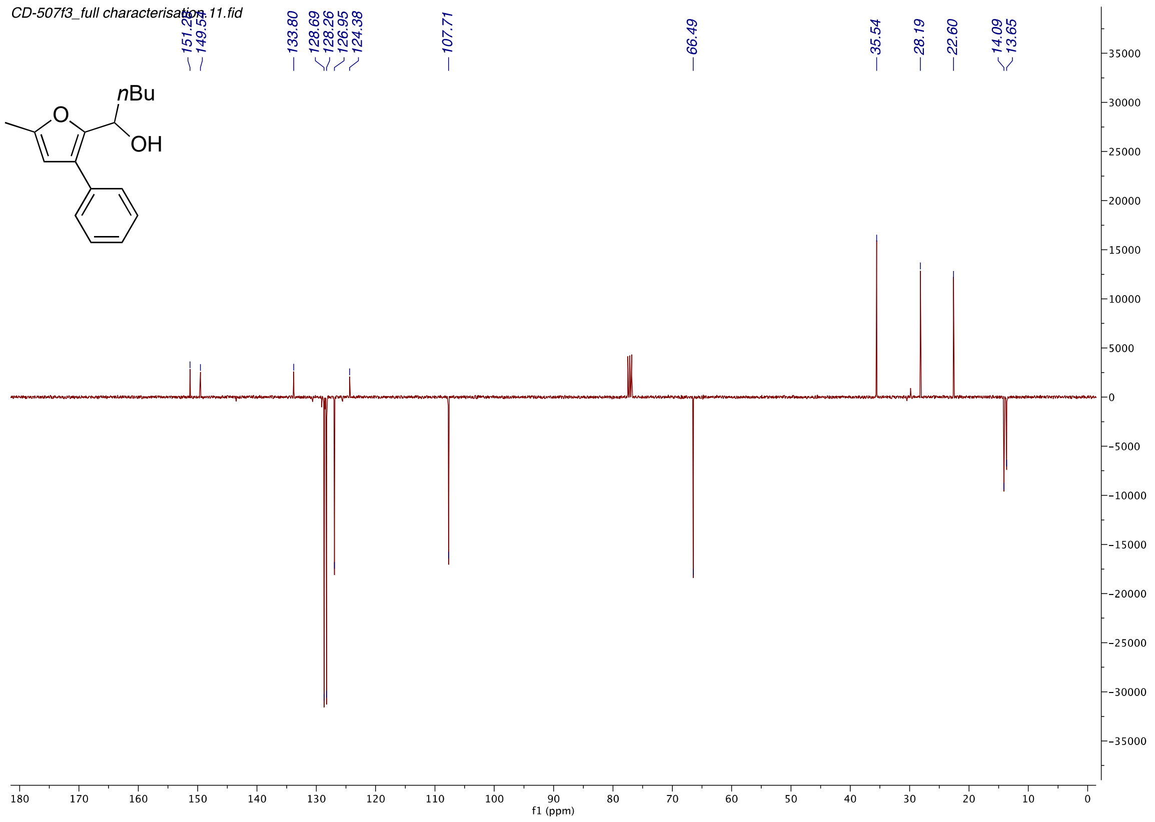

1-(3-(4-methoxyphenyl)-5-methylfuran-2-yl)pentan-1-ol (19)

^{1}H NMR (400 MHz, $CDCl_3$)

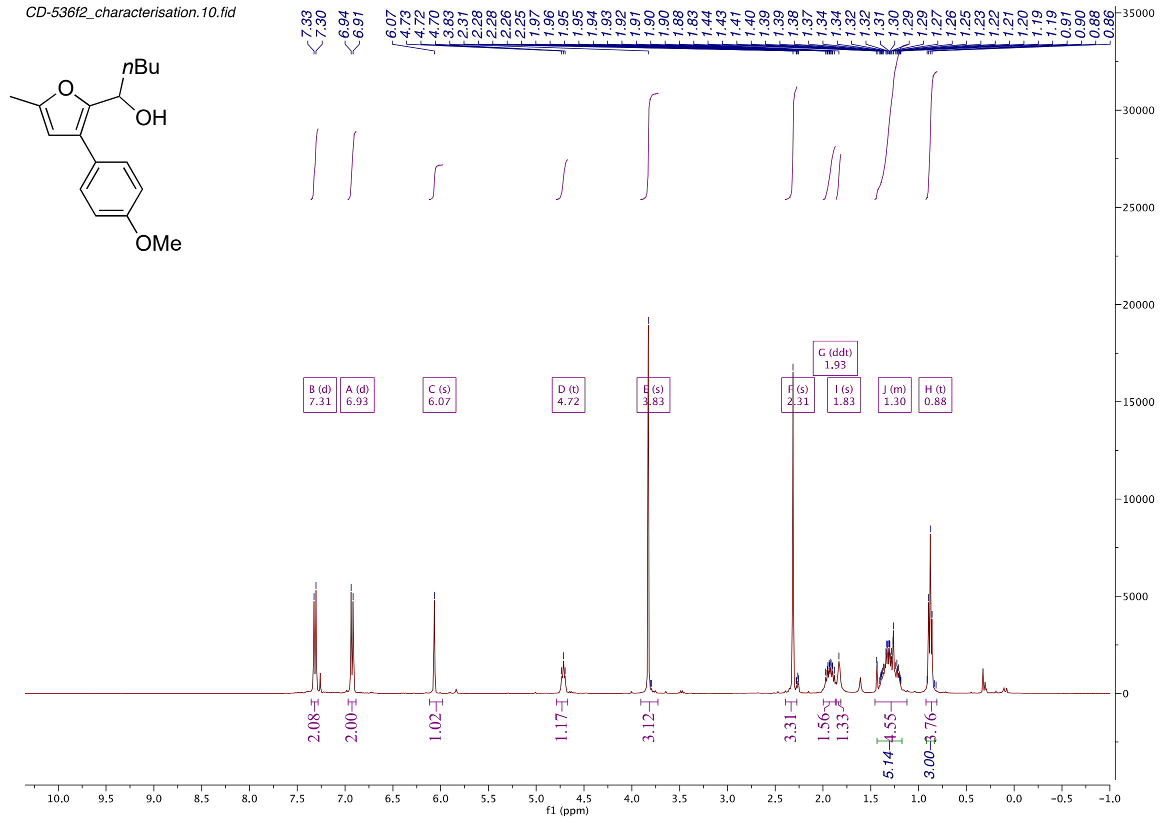

^{13}C Jmod NMR (100 MHz, $CDCl_3$)

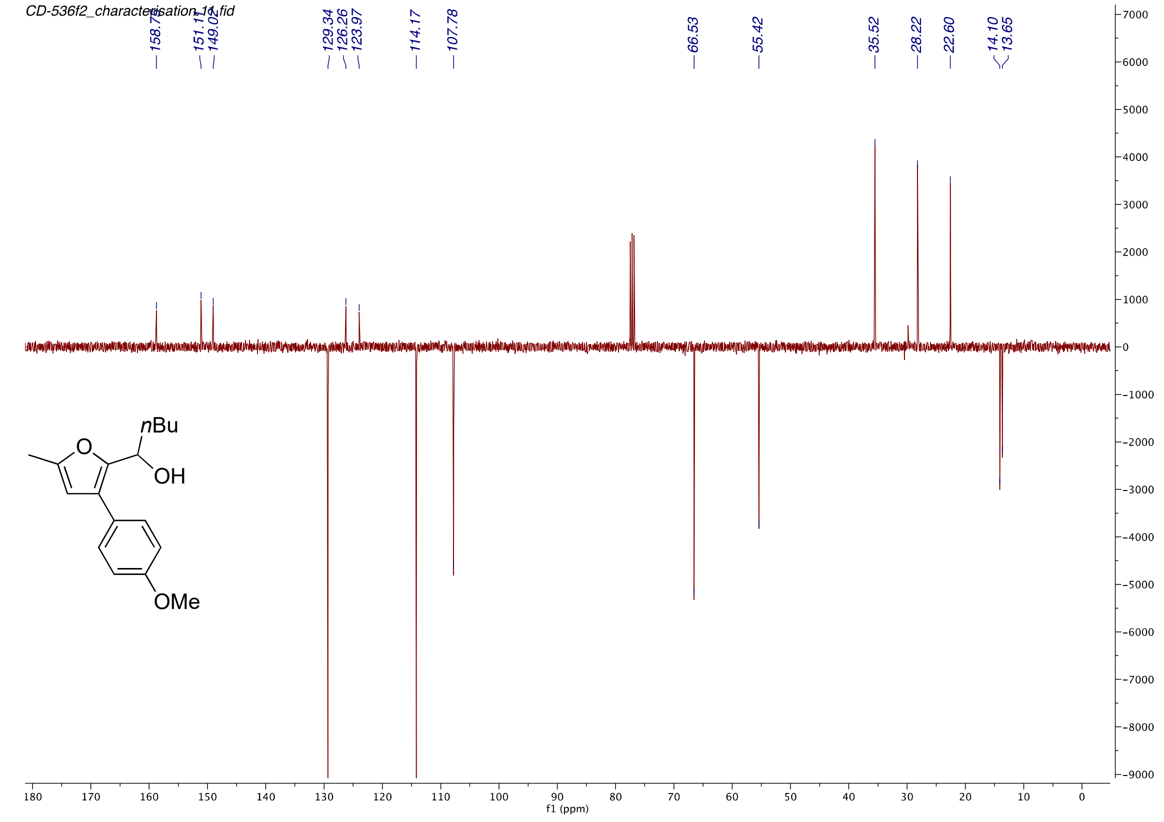

1-(5-methyl-3-(2-methylallyl)furan-2-yl)pentan-1-ol (21)

^{1}H NMR (400 MHz, $CDCl_3$)

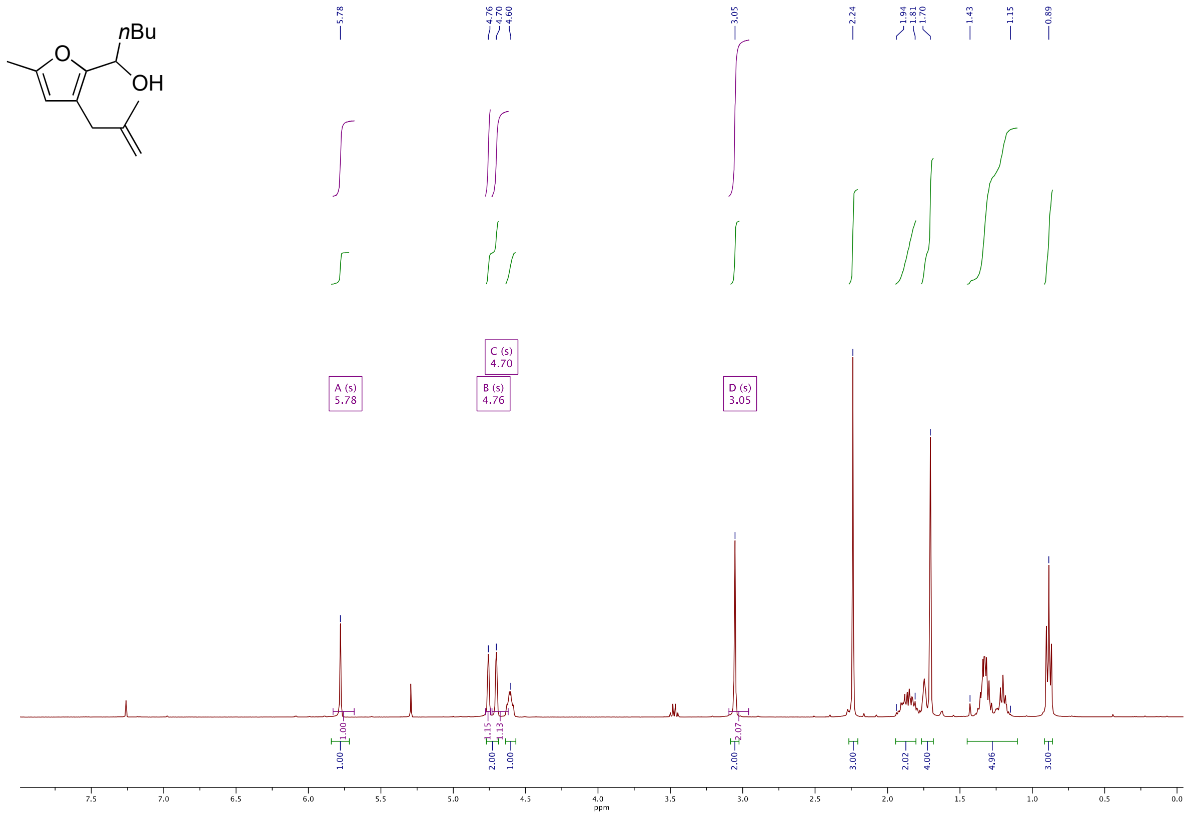

^{13}C Jmod NMR (100 MHz, $CDCl_3$)

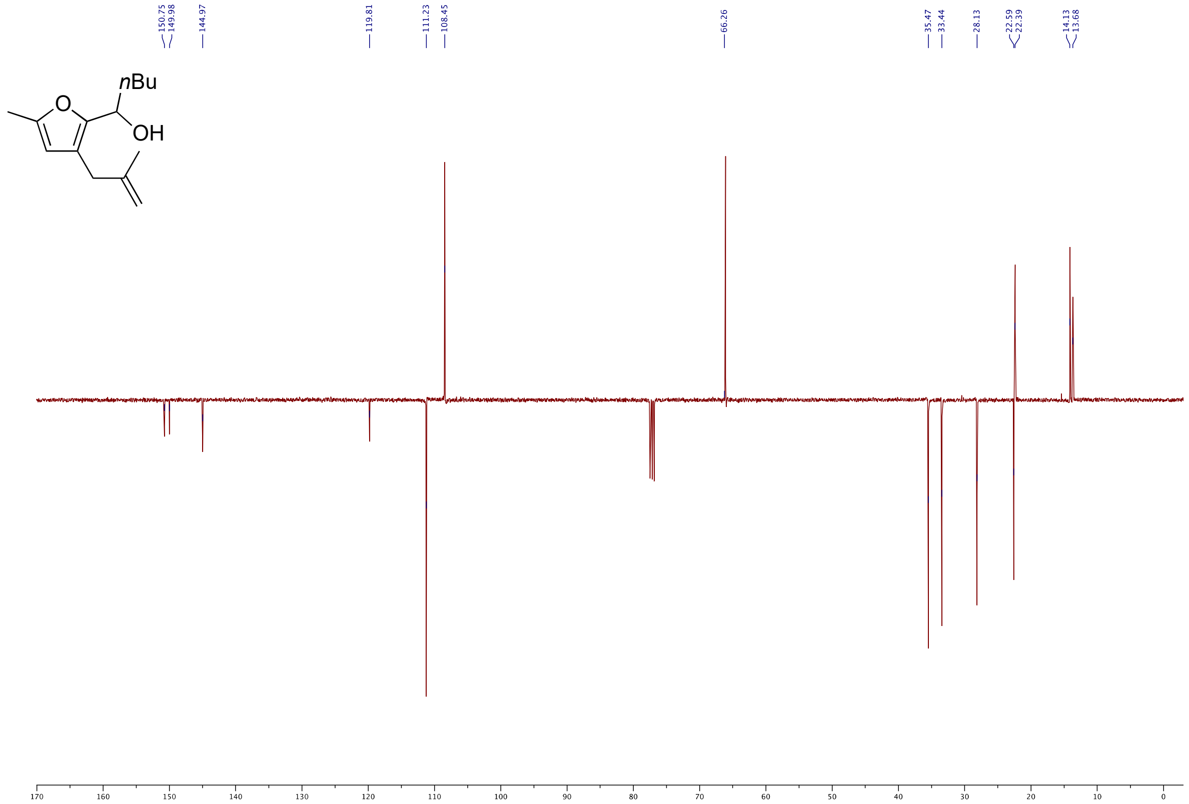

(5-methyl-3-(2-methylallyl)furan-2-yl)(phenyl)methanol (23)

^{1}H NMR (400 MHz, $CDCl_3$)

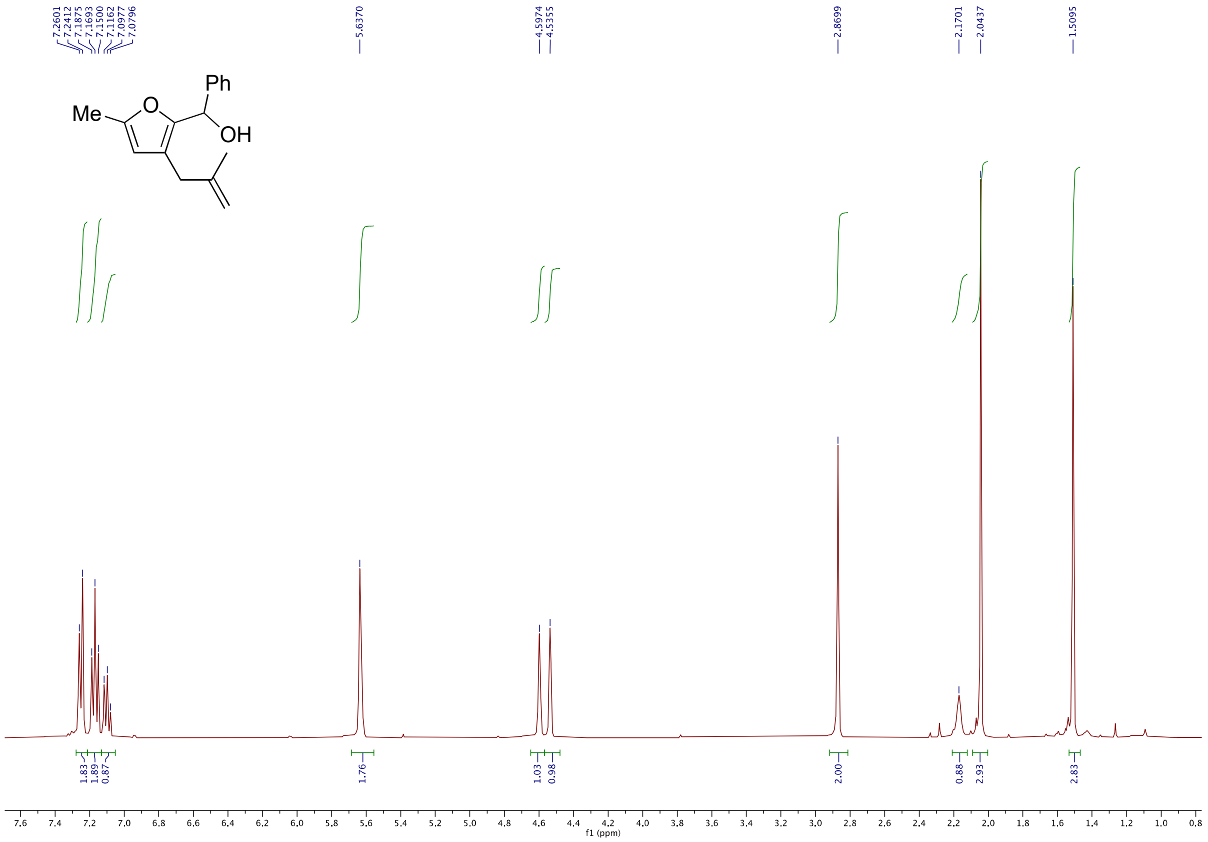

^{13}C Jmod NMR (100 MHz, $CDCl_3$)

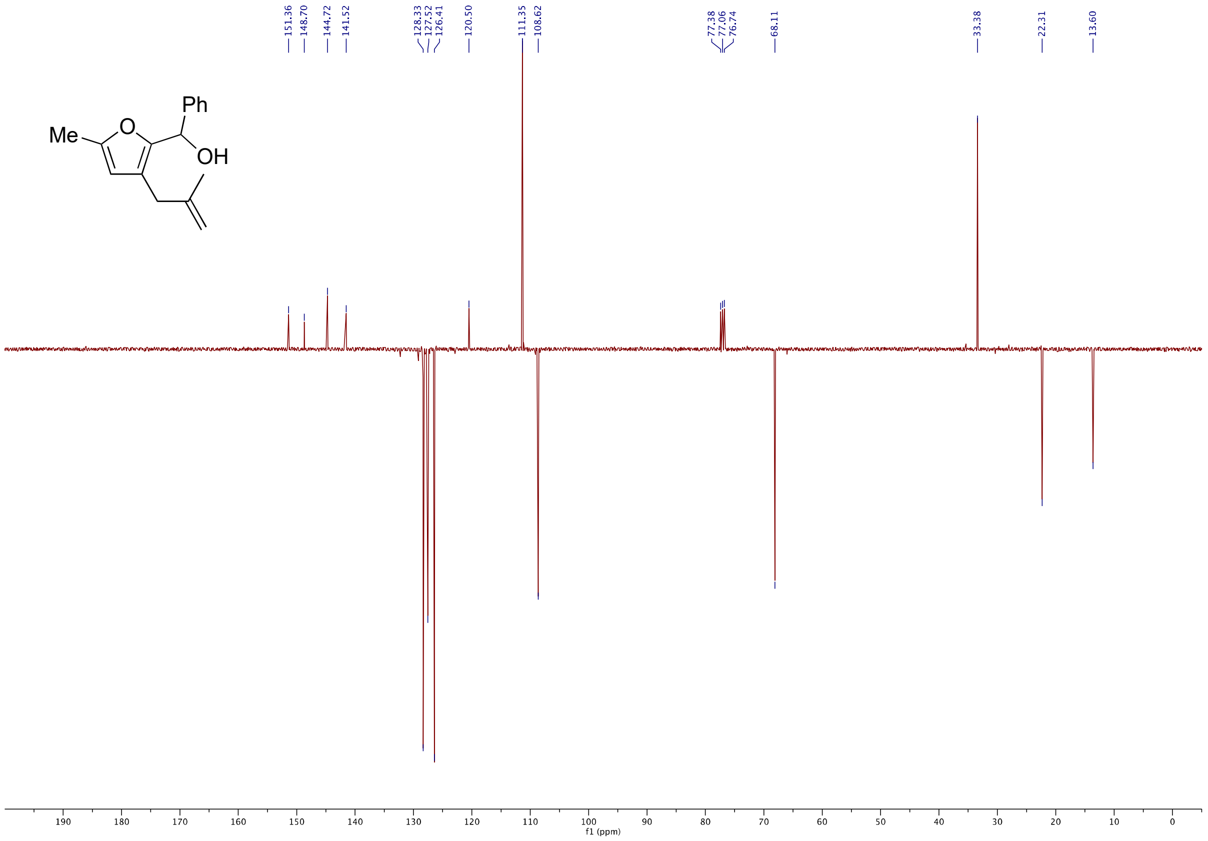

1-(5-methyl-3-(2-methylallyl)furan-2-yl)but-3-en-1-ol (24)

^{1}H NMR (400 MHz, $CDCl_3$)

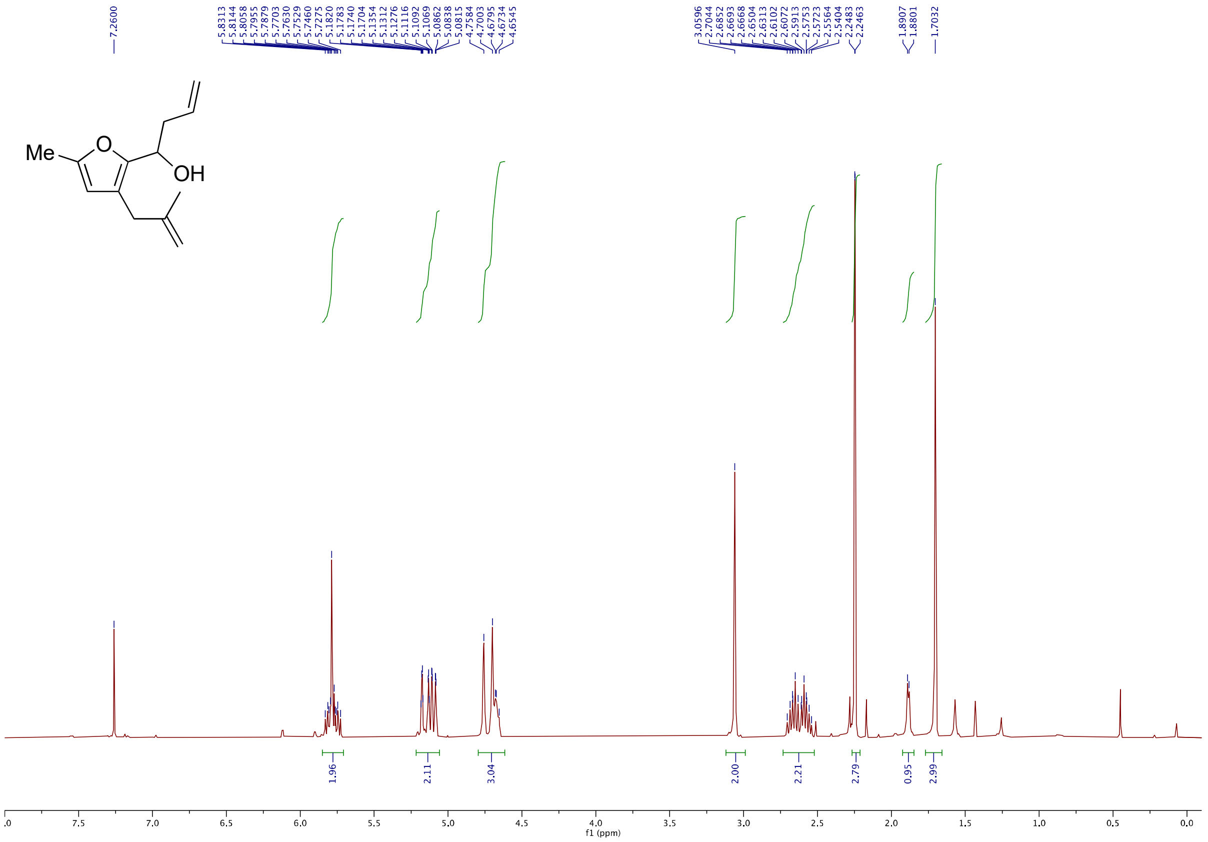

^{13}C Jmod NMR (100 MHz, $CDCl_3$)

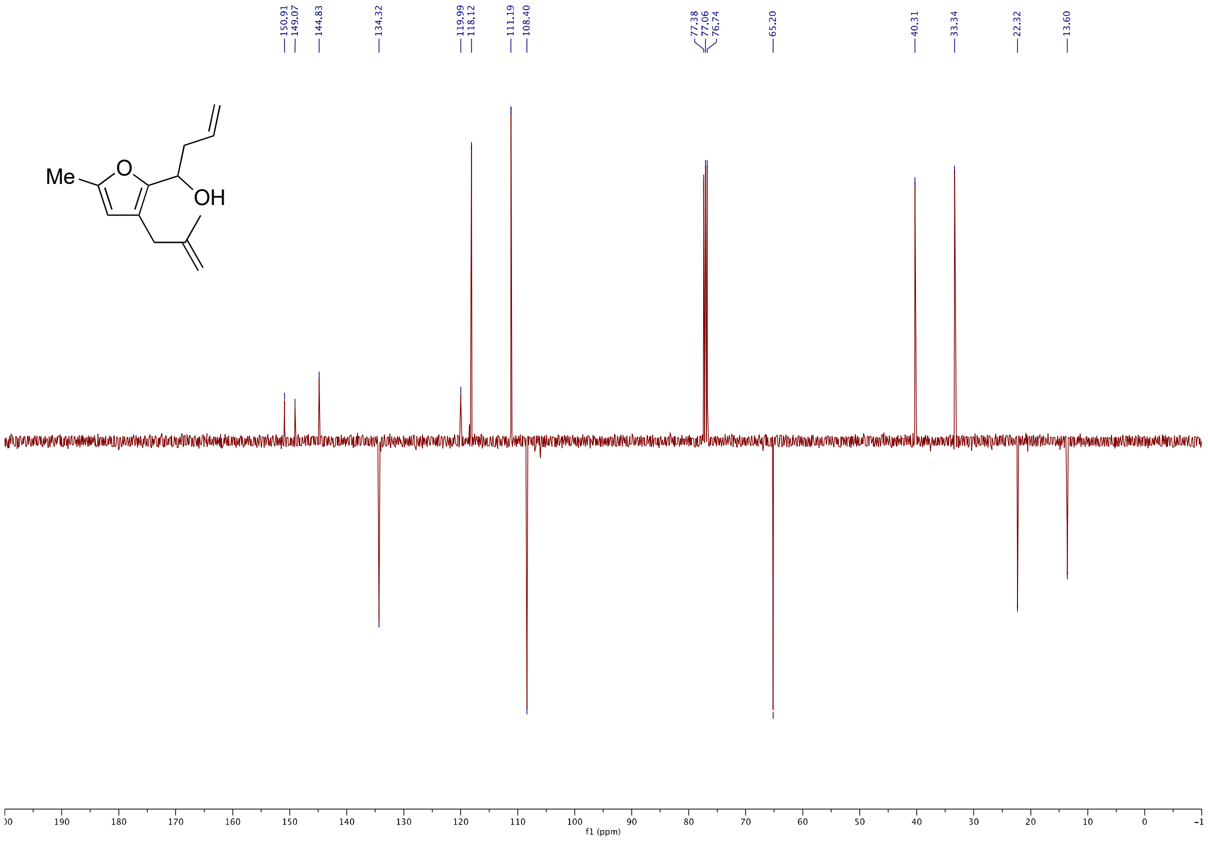

1-(3-(2-methylallyl)furan-2-yl)pentan-1-ol (25) (containing 36% **25'**)

^{1}H NMR (400 MHz, $CDCl_3$)

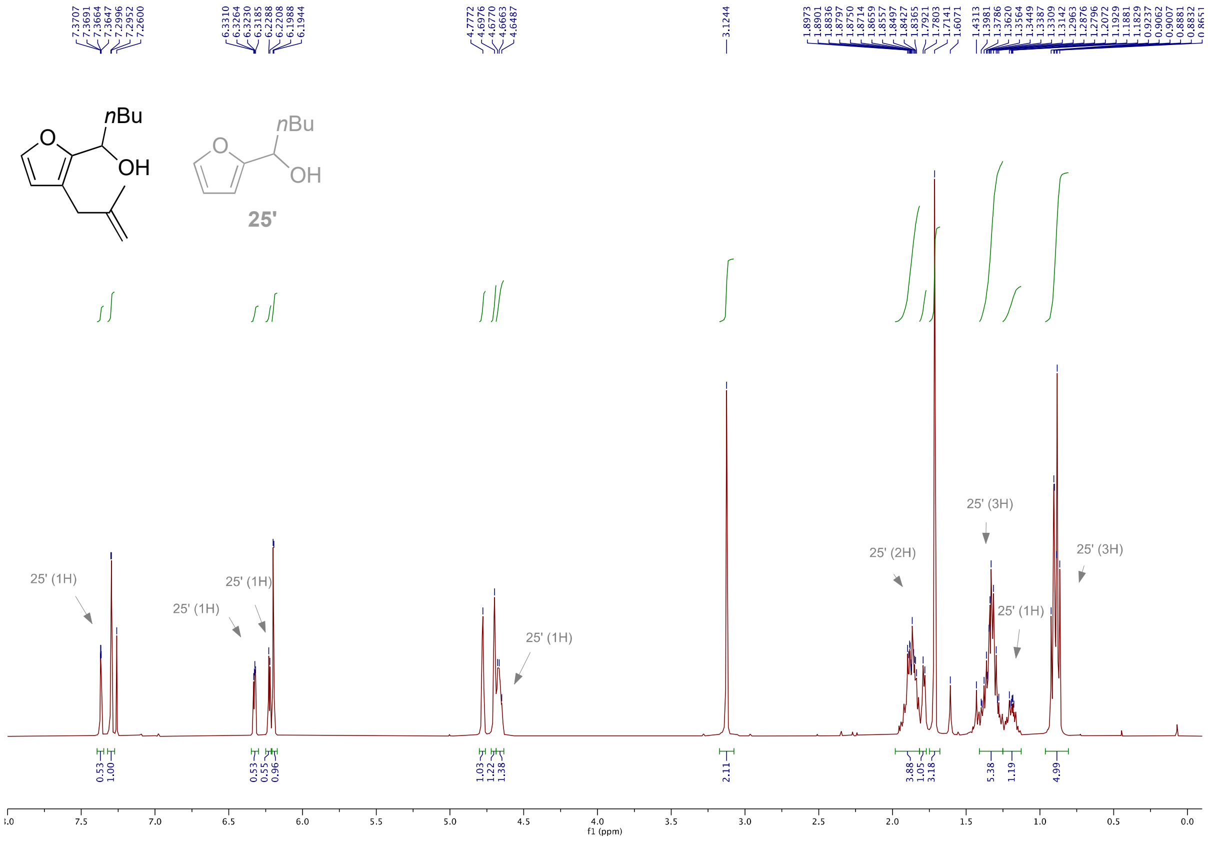

^{13}C NMR (100 MHz, $CDCl_3$)

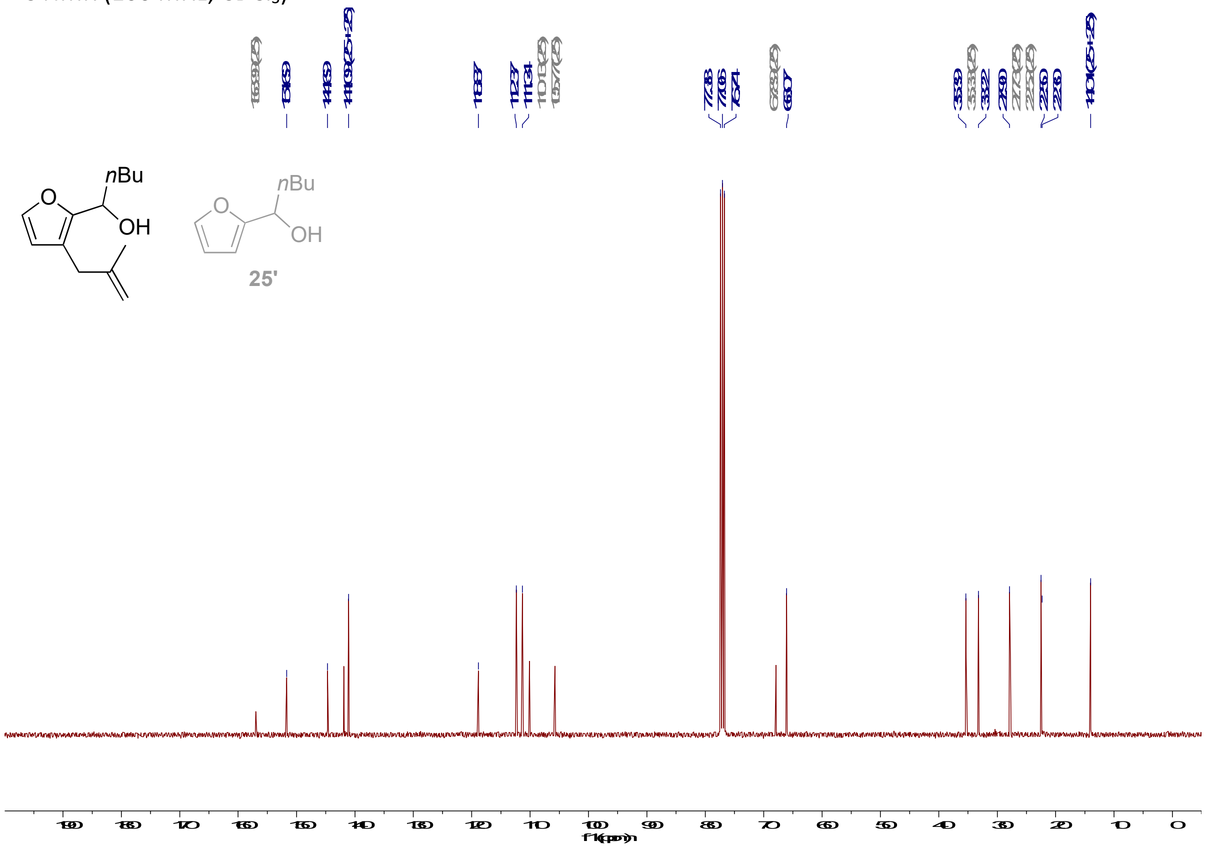

(3-(2-methylallyl)furan-2-yl)(phenyl)methanol (26) (containing 13% **26’**)

^{1}H NMR (400 MHz, $CDCl_3$)

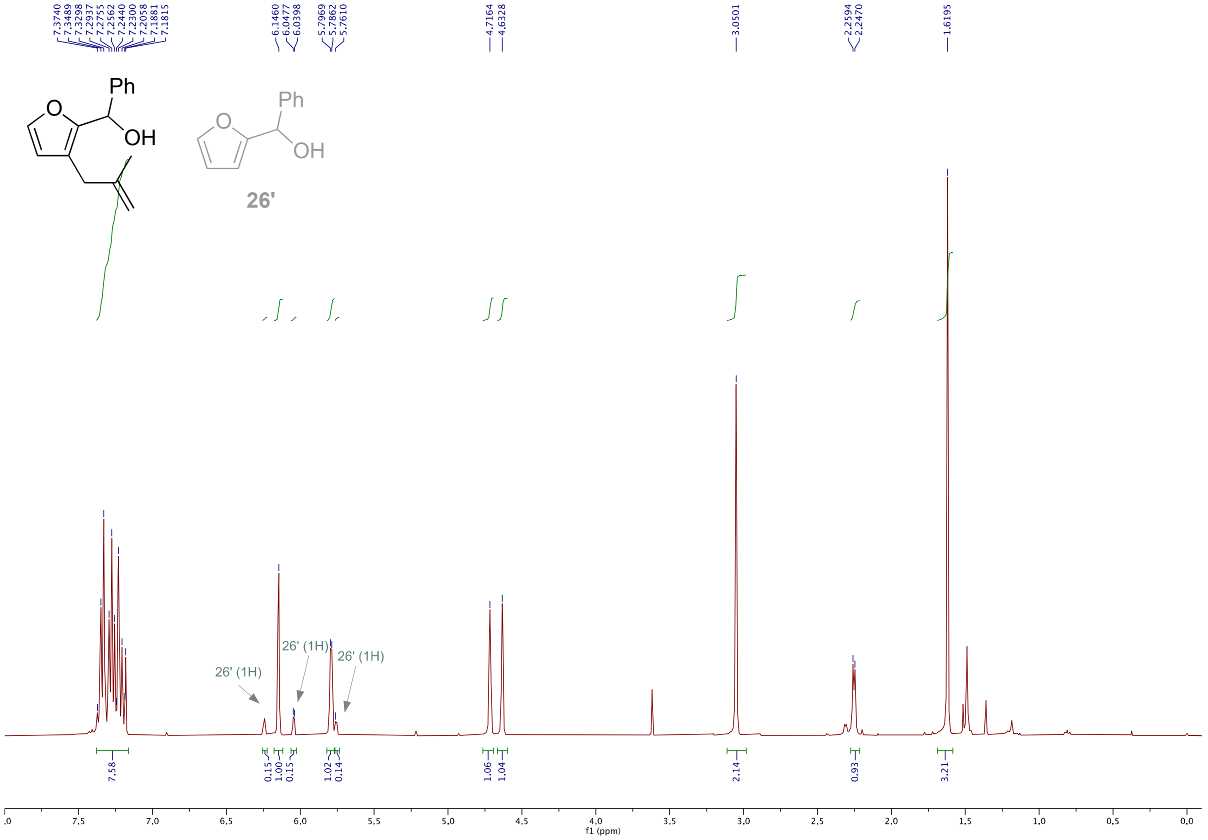

^{13}C Jmod NMR (100 MHz, $CDCl_3$)

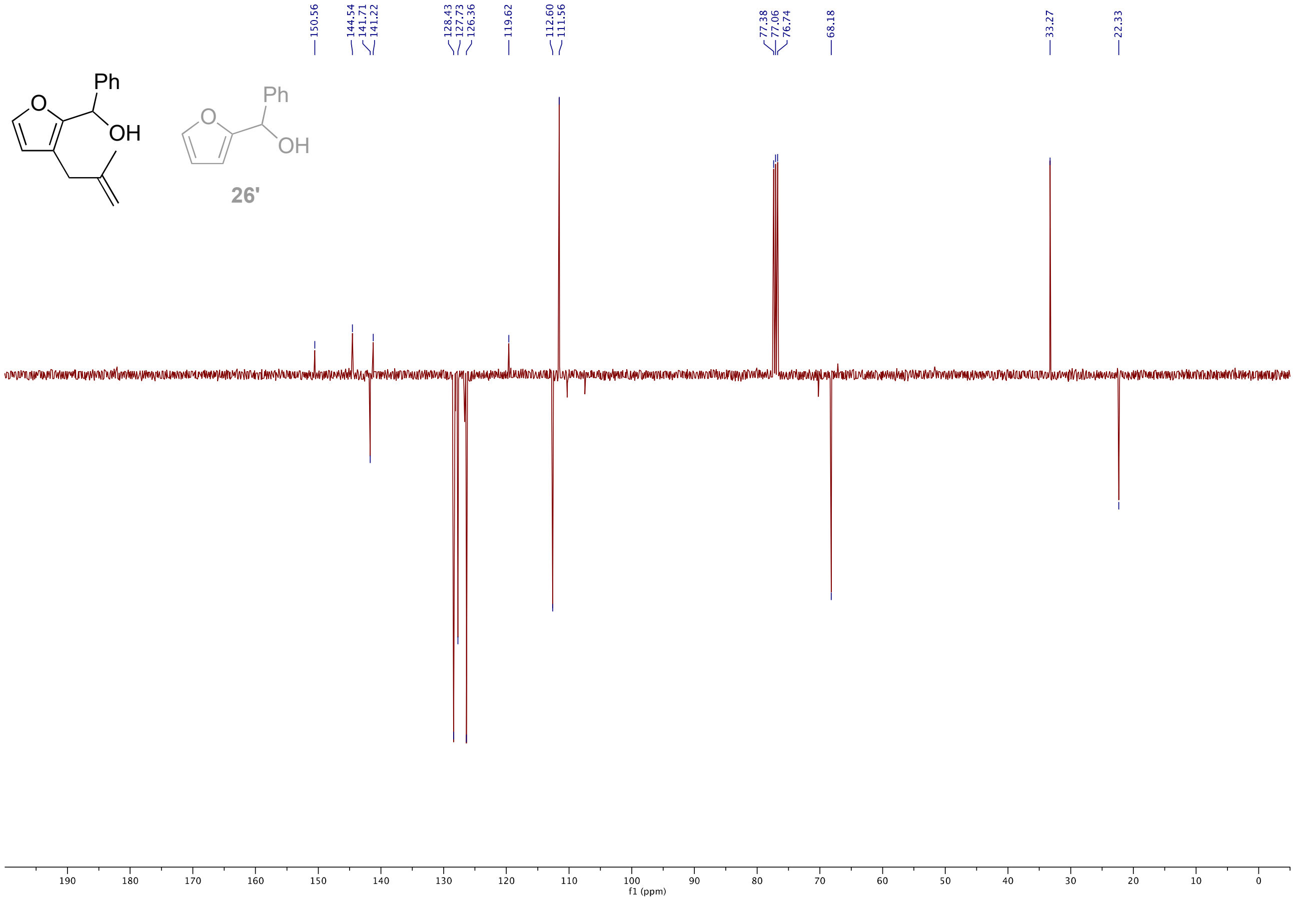

1-(3-allyl-5-methylfuran-2-yl)pentan-1-ol (27) (containing 14% **27’**)

^{1}H NMR (400 MHz, $CDCl_3$)

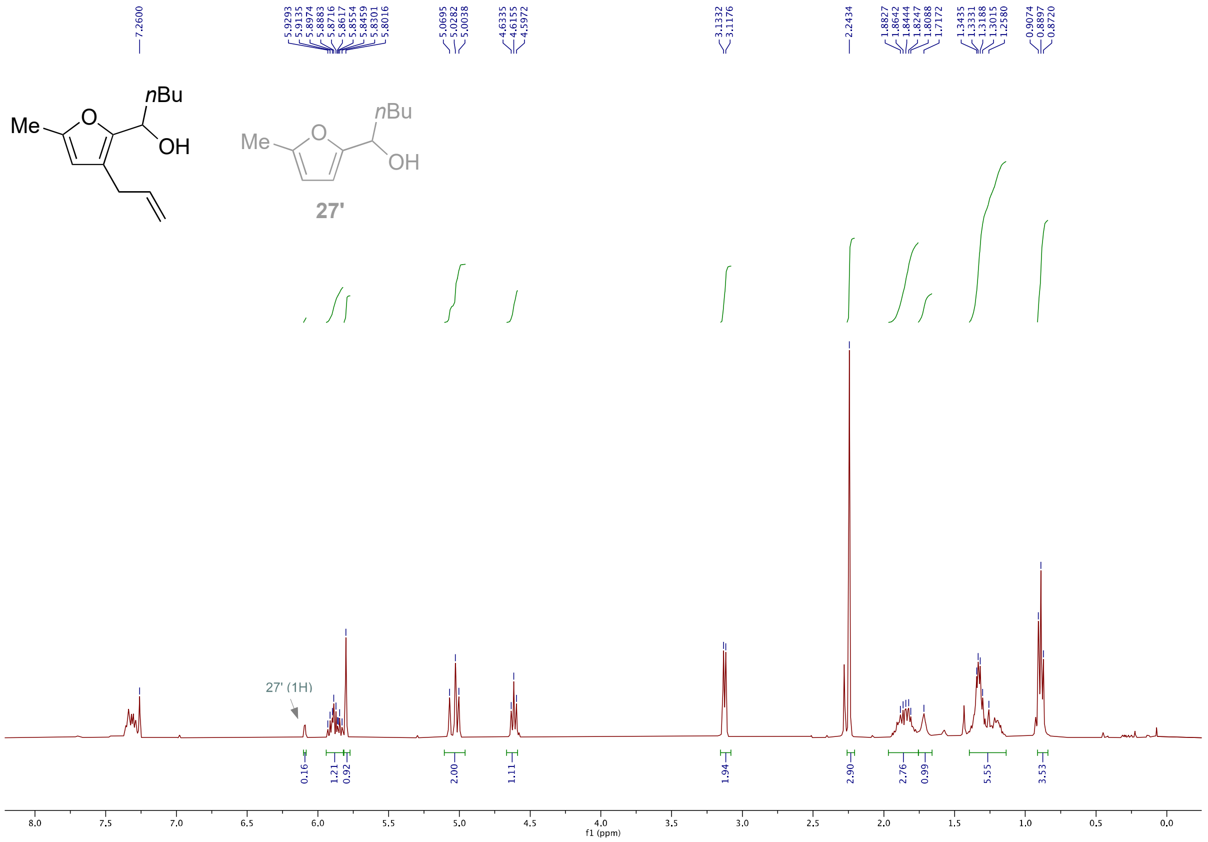

^{13}C Jmod NMR (100 MHz, $CDCl_3$)

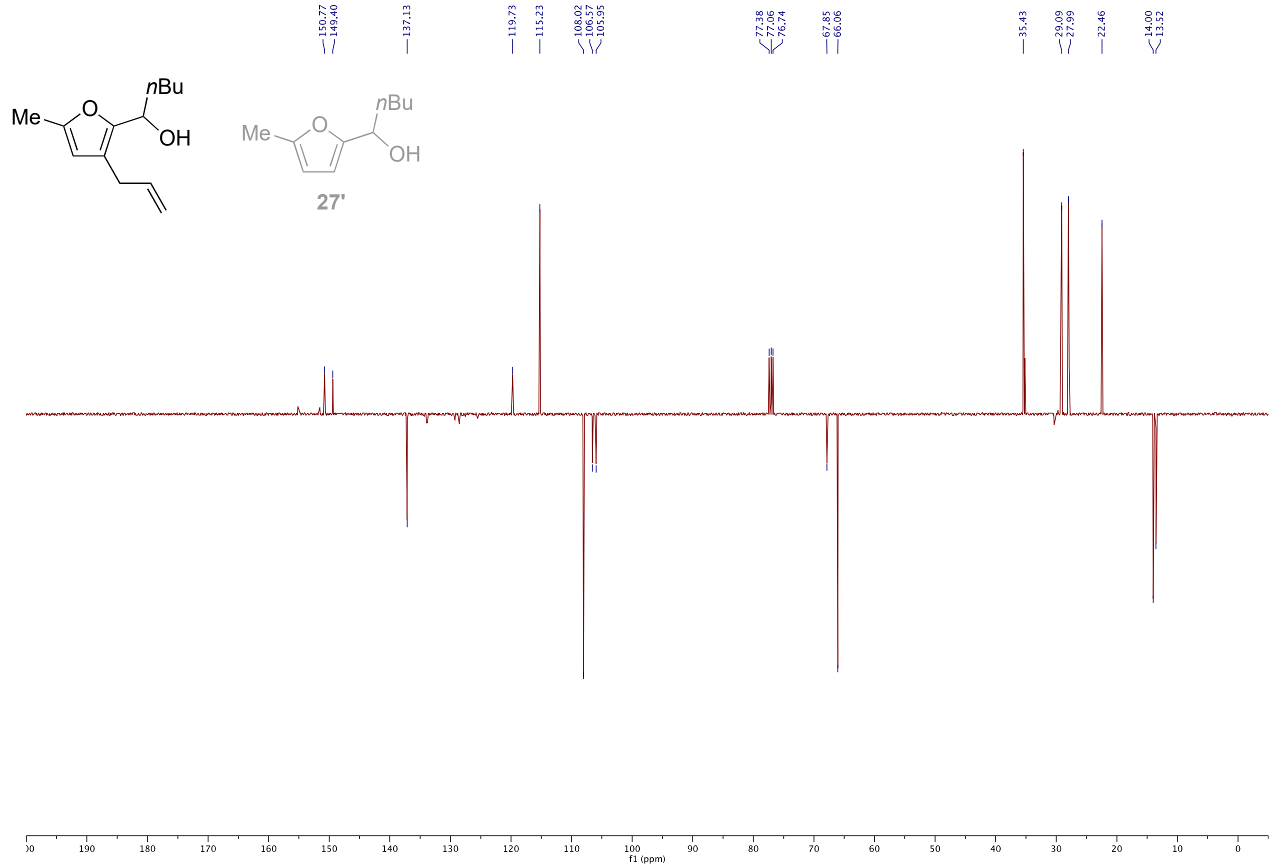

1-(3,5-dimethylfuran-2-yl)pentan-1-ol (28)

^{1}H NMR (400 MHz, $CDCl_3$)

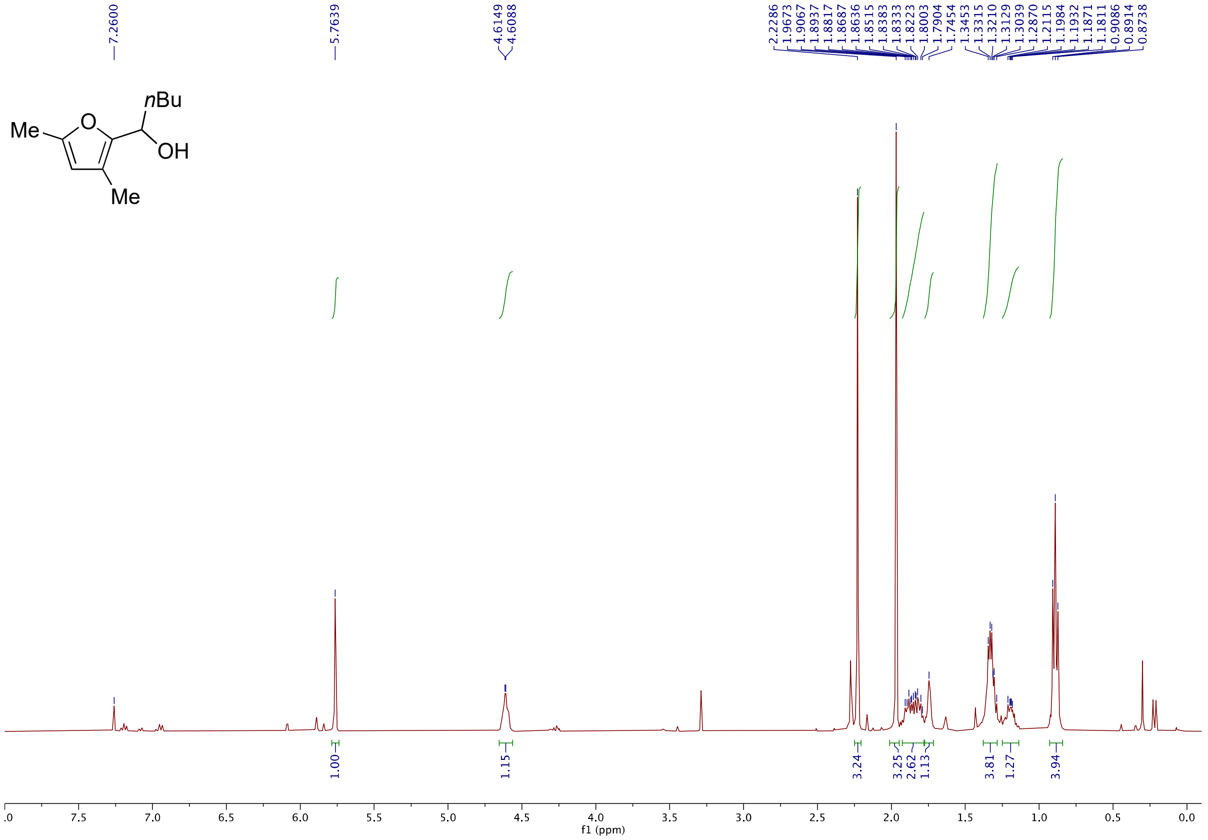

^{13}C Jmod NMR (100 MHz, $CDCl_3$)

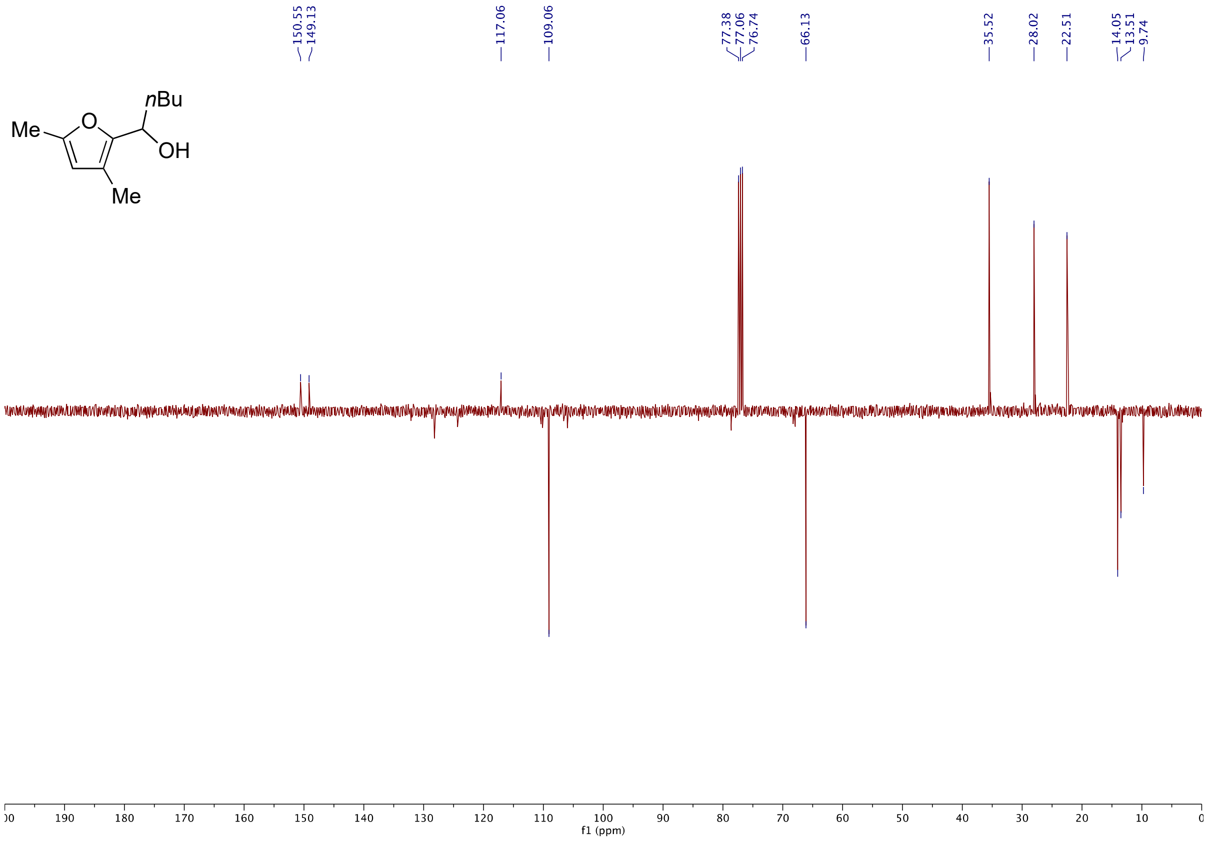

VI. **References**

1 S. Curpanen, G. Poli, A. Perez-Luna, J. Oble, *Asian J. Org. Chem* **2022**, e202200199.
2 D. W. Kim, H.-J. Jeong, S. T. Lim, M.-H. Sohn, *Angew. Chem. Int. Ed.* **2008**, *120*, 8532–8534.
3 Y. Wang, Y. Xiao, T. T. Yang Tan, S.-C. Ng, *Tetrahedron Lett.* **2008**, *49*, 5190–5191
4 F. Saito, S. Iwamoto, R. Yamauchi, *Bioscience, Biotechnology, and Biochemistry* **2010**, *74*, 168– 174.
